# Supplementary material for: Dance for people with chronic respiratory disease: a qualitative study
Source: BMJ Open. 2020 Oct 13;10(10):e038719. doi: 10.1136/bmjopen-2020-038719 (PMC7554453; doi:10.1136/bmjopen-2020-038719)
Supplement: Supplementary data [file bmjopen-2020-038719supp001.pdf]

Title of Project:

Activity, Balance and Chronic Obstructive Pulmonary Disease (ABCOPD) study: Qualitative Dance Component

IRAS Project ID: 258399

Topic Guide for Interviews

Topics listed below were trialled and refined with respiratory specialist patient advisory groups prior to formal data collection.

All recordings should start with a clear statement of study participation consent in addition to the written. Initially discussion should start with rapport building with study participants. All interviews and focus groups will have audio recording on a dictaphone.

Key Topics:

How does having [primary respiratory diagnosis] affect your life?

- Symptoms
- Mood/mental health
- Socially

Physical activity (how much people do) in general life:

- What do you know about physical activity?
- What does it mean to you?
- How physically active are you?
- Has your experience of physical activity changed over time?
  -and living with lung disease?
- Should activity be avoided or actively engaged in?
- What sort of things can be done to improve physical activity levels in general?
  -and for you?

Physical capacity and physical performance (how much people can do and how well they do it)

- How would you describe the quality of your movement (in relation to stability, speed, strength and coordination?)
- Has the quality of your movement changed?
- Has your experience of your movement quality changed, for example do you find balancing, moving, or doing daily tasks more or less challenging than previously?

Experience of dance participation

How long have you been dancing?
Why do you do it?
Tell me about the dance group
How do you feel during the dance sessions?
What do you enjoy?
What do you not enjoy?

Impact of participation

What do you think are the main impacts of participating in the dance group?
Main positives?
Main negatives?
How has dancing impacted on your experience of living with lung disease?
Do you have any ideas for improving the classes?
What would you tell others who have lung disease about dancing?

Any other comments

13-FEB-19 V1
