# Supplementary material for: Dance for people with chronic respiratory disease: a qualitative study
Source: BMJ Open. 2020 Oct 13;10(10):e038719. doi: 10.1136/bmjopen-2020-038719 (PMC7554453; doi:10.1136/bmjopen-2020-038719)
Supplement: Author's manuscript [file bmjopen-2020-038719.draft_revisions.pdf]

BMJ Open

BMJ Open is committed to open peer review. As part of this commitment we make the peer review history of every article we publish publicly available.

When an article is published we post the peer reviewers' comments and the authors' responses online. We also post the versions of the paper that were used during peer review. These are the versions that the peer review comments apply to.

The versions of the paper that follow are the versions that were submitted during the peer review process. They are not the versions of record or the final published versions. They should not be cited or distributed as the published version of this manuscript.

BMJ Open is an open access journal and the full, final, typeset and author-corrected version of record of the manuscript is available on our site with no access controls, subscription charges or pay-per-view fees (http://bmjopen.bmj.com).

If you have any questions on BMJ Open's open peer review process please email info.bmjopen@bmj.com

Dance for people with chronic respiratory disease: A qualitative study

Journal:	*BMJ Open*
Manuscript ID	bmjopen-2020-038719
Article Type:	Original research
Date Submitted by the Author:	20-Mar-2020

Complete List of Authors:	Philip, Keir; National Heart and Lung Institute,<br>Lewis, Adam; Brunel University London<br>Williams, Sian; National Heart and Lung Institute<br>Buttery, Sara; National Heart and Lung Institute<br>Polkey, Mike; Royal Bromton and Harefield NHS Trust,<br>Man, William; Royal Brompton & Harefield, Respiratory Medicine<br>Fancourt, Daisy; University College London Research Department of Epidemiology and Public Health, Department of Behavioural Science and Health<br>Hopkinson, Nicholas; Imperial College London, National Heart and Lung Institute

Keywords:	Adult thoracic medicine < THORACIC MEDICINE, Chronic airways disease < THORACIC MEDICINE, REHABILITATION MEDICINE, COMPLEMENTARY MEDICINE

SCHOLARONE™
Manuscripts

I, the Submitting Author has the right to grant and does grant on behalf of all authors of the Work (as defined in the below author licence), an exclusive licence and/or a non-exclusive licence for contributions from authors who are: i) UK Crown employees; ii) where BMJ has agreed a CC-BY licence shall apply, and/or iii) in accordance with the terms applicable for US Federal Government officers or employees acting as part of their official duties; on a worldwide, perpetual, irrevocable, royalty-free basis to BMJ Publishing Group Ltd ("BMJ") its licensees and where the relevant Journal is co-owned by BMJ to the co-owners of the Journal, to publish the Work in this journal and any other BMJ products and to exploit all rights, as set out in our licence.

The Submitting Author accepts and understands that any supply made under these terms is made by BMJ to the Submitting Author unless you are acting as an employee on behalf of your employer or a postgraduate student of an affiliated institution which is paying any applicable article publishing charge ("APC") for Open Access articles. Where the Submitting Author wishes to make the Work available on an Open Access basis (and intends to pay the relevant APC), the terms of reuse of such Open Access shall be governed by a Creative Commons licence – details of these licences and which Creative Commons licence will apply to this Work are set out in our licence referred to above.

Other than as permitted in any relevant BMJ Author's Self Archiving Policies, I confirm this Work has not been accepted for publication elsewhere, is not being considered for publication elsewhere and does not duplicate material already published. I confirm all authors consent to publication of this Work and authorise the granting of this licence.

For peer review only

Dance for people with chronic respiratory disease: A qualitative study

Authors: Keir EJ Philip[1] *, Adam Lewis[2], Siân Williams[1], Sara C Buttery[1], Michael I Polkey[1,3], William Man[3], Daisy Fancourt[4], Nicholas S Hopkinson[1]

Affiliations:

1) National Heart and Lung Institute, Imperial College London
2) Brunel University London
3) Respiratory Medicine, Royal Brompton and Harefield NHS Foundation Trust
4) Department of Behavioural Science and Health, University College London

*Corresponding author

Author Email Addresses

- Keir Philip: k.philip@imperial.ac.uk
- Adam Lewis: Adam.Lewis@brunel.ac.uk
- Siân Williams: sian.health@gmail.com
- Sara Buttery: s.buttery@rbht.nhs.uk
- Michael Polkey: m.polkey@rbht.nhs.uk
- William Man: w.man@rbht.nhs.uk
- Daisy Fancourt: d.fancourt@ucl.ac.uk
- Nicholas Hopkinson: n.hopkinson@imperial.ac.uk

Key Words: respiratory, dance, breathlessness, physical activity, rehabilitation.

Manuscript word count: 3691 (including quotes)

For peer review only

For peer review only - http://bmjopen.bmj.com/site/about/guidelines.xhtml

Abstract

Objectives: To explore the experiences and perceived impact on health and well-being related to participation in a dance group for people with chronic respiratory disease (CRD).

Design: Qualitative study using thematic analysis of semi-structured interviews.

Setting: A community dance group in a UK health centre.

Participants: Convenience sample of long-term dance group participants.

Intervention: Weekly community dance sessions designed for people with breathlessness, lasting 75 minutes, led by a trained community dance leader.

Results: Convenience sample of eight participants, 6 females, aged 57 to 87 years (mean 75), with a median 2-year attendance at weekly dance sessions. Long-term attendance was driven by strongly held beliefs regarding the health and well-being benefits of participation. Four key themes were identified: Dance as (1) a holistically beneficial activity, with physical and psychosocial health benefits including improved or maintained physical fitness and psychological well-being, and reduced need for healthcare; (2) An integral part of their life; (3) An enjoyable activity; and (4) a source of deep social cohesion.

Conclusions: Dance group participants perceived a broad range of health benefits of relevance to the biopsychosocial impacts of their respiratory disease. The themes identified are useful in the ongoing planning and evaluation of dance as a holistic complex intervention for people with CRD, and other arts-based interventions. Further research is required to assess the extent of health impacts identified, and how dance might be most effectively placed as an option in the management of CRD.

Registered as a component of the ABCOPD study at the NIH U.S. National Library of Medicine ClinicalTrials.gov: https://clinicaltrials.gov/ct2/show/NCT04006015?cond=ABCOPD&draw=2&rank=1

For peer review only

Article Summary: Strengths and Limitations of this study

- The first study of long-term real-world participants of a community dance group for people with chronic respiratory disease and breathlessness.
- In depth exploration of participant experience.
- The sample was from a single group which may limit transferability.
- Semi-structured interviews facilitated a depth of understanding of clinically relevant topics, aiding the potential application of findings

Original Protocol/topic guide

See attached (supplementary file)

Funding statement

KP was supported by the Imperial College Clinician Investigator Scholarship. DF was supported by the Wellcome Trust [205407/Z/16/Z]. The funders had no say in the design and conduct of the study; collection, management, analysis, and interpretation of the data; preparation, review, or approval of the manuscript; and decision to submit the manuscript for publication.

Competing Interests

Siân Williams is the founder of the dance group and leads the sessions. She was not involved in the thematic analysis.

No other conflicts of interest declared.

Authors Contributions

KP had the original idea for the study. KP and AL wrote the first draft of the manuscript. All authors contributed to the study design, writing, reviewing and editing the manuscript.

Reporting Checklist

See attached COREQ checklist.

Data sharing statement

Data from this study are not being made available for sharing as it would not be possible to anonymise the interview transcripts given the personal content included and the very limited number of Dance groups for respiratory conditions in London.

Patient and Public Involvement

Patients and public were not involved in the design of the study, however the study specifically focuses on patient/participant perceptions.

For peer review only

Introduction

Chronic respiratory diseases (CRD) are leading causes of global morbidity and mortality (1), often causing persistent and disabling symptoms including breathlessness, cough, skeletal muscle impairment and exercise limitation (2), as well as psychosocial impacts such as depression, loneliness and social isolation (3, 4). Physical activity, exercise training and pulmonary rehabilitation (PR) are core components of CRD management (5, 6). However, engagement is inhibited by symptoms, availability, accessibility and attractiveness of local options (7, 8). Recently, interest in alternative forms of physical activity and exercise training has increased including Tai Chi (9, 10), Yoga (11) and Singing for Lung Health (SLH) (12-16). Furthermore, the current focus on social prescribing means such approaches are of increasing interest (17). Though an extensive body of research suggests arts interventions can improve health and wellbeing (18, 19), respiratory specific arts-in-health research is currently limited. Nevertheless evidence which exists suggests a range of biopsychosocial benefits (20).

Dance has been shown to be beneficial in healthy people and individuals with a range of medical conditions. Impacts include improved physical performance, function, mood, and social engagement, which are all highly relevant for people with CRD (21-28). Furthermore, dance is a core aspect of societies and cultures globally, hence has great potential as an enjoyable, engaging intervention in respiratory care throughout the world (29, 30). Regarding dance for people with CRD, two feasibility studies found dance for CRD interventions to be feasible (31, 32), one also suggested dance may improve functional capacity, balance, anxiety and depression, physical activity, and health-related quality of life (32). However, no studies have assessed participants' experiences and perceptions regarding health and wellbeing impacts, which is important given the need to understand the potential of dance for wider application and how to implement and evaluate it in trials and clinical practice.

The objective of this study was to explore the experiences and perceived health and well-being impacts of participation in a community-based dance group for people with CRD - 'Dance Easy'.

Method

Research design

We performed a qualitative study using thematic analysis within a critical realist paradigm, to explore the perceptions of participants of a community dance group for people with CRD. The primary focus was their views on the impact of participation on their health and wellbeing.

We used semi-structured interviews (see topic guide appendix), developed by reviewing research on the impacts of conceptually related interventions such as PR, SLH and Tai-Chi for people with CRD. This contributed deductive elements to the analysis. However, analysis was primarily inductive using open-ended questions and open coding. Participants were encouraged to continue discussing topics which appeared meaningful with prompts. Interviews were complemented by structured observations by KP, who participated by dancing with the group over 4 sessions. This participation aimed to build rapport and trust with participants to facilitate interviews, and make structured observations to contextualise interview content, and triangulate themes. Additionally, this method enabled greater reflexivity within the analysis. Participants were made aware that KP is a clinician (but not involved in their clinical care) with experience of community dance groups.

For peer review only - http://bmjopen.bmj.com/site/about/guidelines.xhtml

The eight regular group participants, were approached face-to-face by the group leader, and all consented to participate (convenience sample) in the study. Eligibility criteria included a clinical diagnosis of CRD, age >18 years and able to consent. in All participants received participant information sheets and provided signed informed consent.

Dance Group intervention

The group started in May 2017, with seven of the study participants present from the first few months. The group meets weekly in a community centre attached to a health centre in London. Participants usually meet in the café from 30 minutes before the session. The sessions are free for participants, the leader volunteers her time for free, and the space is provided by Whittington Health.

Sessions last approximately 75 minutes and include a warmup, including qigong movements, progressively complex group dance routines, warm-down, stretching and relaxation. The session leader (SW) is a trained community dance group leader with extensive experience working with people with CRD. Music is played throughout the sessions and includes an eclectic mix of genres, selected by the session leader and participants, to inspire dancing but not feel too fast. Each track corresponds to a specific movement routine. Dances are selected from a repertoire, with new dances added gradually. Though led by SW, participants contribute their opinions, movements and artistic expressions.

The dances are sufficiently physically demanding to induce breathlessness, though not excessively. Given the group's heterogeneity regarding disease severity, age, and co-morbidities, a degree of personalisation is required. This is achieved by providing optional variations on movements, and allowing participants to pace themselves and take breaks as required. Sessions are structured yet relaxed, focusing on dance as an enjoyable activity. Short breaks between dances are included for recovery and are used creatively to talk about dance, music and other topics that emerge. After the dance session, four to six participants go to a local pub to eat and chat, which developed spontaneously and became established.

Data collection

Convenience sampling was used. Interviews were conducted one-to-one, by KP (see researcher info). Reflections and impressions were documented immediately following the interviews, with memo writing continuing throughout the analytic process. Interviews were conducted privately in the room where the dance takes place, or the café, depending on participant preference. Interviews, recorded by dictaphone, lasted 24 to 43 minutes, and consisted of open-ended questions, semi-structured around the following topics:

- Experience of respiratory illness and impact on life
- Experience of dance group
- Perceived impacts of dance group participation

Data analysis

Interviews were transcribed verbatim by KP. KP and AL conducted thematic analysis as outlined by Braun and Clarke (33). Interviews were transcribed verbatim by KP, then KP and AL conducted further analysis. During phase 1, transcripts were read and re-read, and audio recordings listened to repeatedly. In phase 2, open inclusive coding of transcripts was used. This generated first cycle codes, mainly in-vivo (verbatim), descriptive, process, emotive and value items. Second cycle coding used the context from the first cycle, then focused on discussions relating to dance, activity and their disease. Code mapping facilitated code clustering into meaningful associated units. Preliminary themes (phase

For peer review only - http://bmjopen.bmj.com/site/about/guidelines.xhtml

3) were constructed from code mapping and reorganisation in relation to the associated quotes. Phases 1-3 were completed by KP and AL independently, before coming together to alter, refine, consolidate and name themes (phase 4 and 5). Structured observations, reflexive accounts post-interview, and memos were incorporated to the thematic analysis. Themes were reviewed with the group leader SW. Participant respondent validation was not performed because of the value placed on researchers' interpretation within our methodology.

Demographic and disease specific information was sought (see table 1). Breathlessness was to indicate severity, rather than lung function measures as breathlessness relates more closely to patient related outcomes including mortality and functional impairment (2). Furthermore, participants could provide this themselves, whereas hospital investigations were not readily accessible and participants had different CRD, which limits the interpretation of spirometry group averages.

Results

Eight regular dance group participants were recruited, representing the group at that time, including 6 women and 2 men, aged 57-87 years (mean 75). All reported CRD, primary diagnoses were reported as COPD (x5), asthma (x2), bronchiectasis (x1), however multimorbidity was common (see table 1).

Table 1: Participant Characteristics

Pt #	Interview (min/s)	Age	Gender	Diagnosis + comorbidities	Duration of attendance	MRC Breathlessness	Use of oxygen therapy	House/flat. Cohabiting?	Previous Pulmonary Rehabilitation
1	26.10	68	F	Asthma, Arthritis	2 years	2	N	House, lives alone	No
2	23.47	84	M	COPD, Type 2 Diabetes Mellitus, arthritis, hip replacement	2 years	2	N	House. Lives alone.	No
3	34.54	68	M	COPD, Emphysema, Atrial Fibrillation	2 years	1	N	Flat. Lives with wife.	Yes
4	24.53	82	F	COPD, asthma, emphysema, aspergillus, A1ATD, hemochromatosis, Osteoarthritis, glaucoma	2 years	2	N	House. Lives with son.	Yes
5	25.57	76	F	COPD	2 years	1	N	House. Lives alone, with dog	No
6	34.42	57	F	COPD, asthma, sarcoidosis	2 years	2	N	Maisonette. Lives with daughter	Yes

For peer review only - http://bmjopen.bmj.com/site/about/guidelines.xhtml

7	43.14	87	F	COPD, emphysema	2 years	3	N	House, lives alone	Yes
8	24.05	80	F	Bronchiectasis	7 months	1	N	House, lives alone	No

No adverse events were reported. Participants perceived a wide range of health and wellbeing benefits related to attendance. All were extremely positive about the group, none expressed significant negatives, though some experienced transport related challenges. Attendance was very good, both self-reported and according to the leader's register. Non-attendance was related to holidays, transport difficulties or health issues such as optician appointments. Participants showed marked determination regarding being physically active so that '*psychologically the illness has not taken over*' (participant 5). They perceived sedentarism as highly negative, often stating the only choice being to '*get on with it*' (participant 2). This determination and commitment was driven by the identified themes, which were dance as (1) A holistically beneficial activity; (2) An important part of their lives; (3) An enjoyable activity; and (4) A source of deep social cohesion. Themes 3 and 4 were drivers of themes 1 and 2, but also stood alone so were not considered subthemes.

Figure 1: Thematic Map

Dance as a holistically beneficial activity

Participants viewed the sessions as a combined exercise, dance, and social activity. They described physical, psychological and social benefits, which were synergistic and, largely inseparable from one another.

> 'it's a bit of everything, afternoon social activity, because everyone has tea when they finish, or pub. It's a bit of exercise, a bit of, entertainment, it's a bit of, overall, learning.' (participant 6)

Participation was perceived as beneficial for overall health status - *'I feel like it's been a great benefit for me. It has impacted on me.'* (participant 3). Impacts were described as improvements *'you're able to do things now'* (participant 3), or physical and cognitive maintenance in the context of ageing:

> '…keeping one's body and one's mind active is very important. And that is really the be all of the end all. That's the object of the exercise' (participant 2), 'it helps you maintain a standard as it were, of operation in the world I suppose' (participant 2)
>
> And
>
> 'Also when the music comes on, you, about three beats and you're like, 'oh', so I like that because you're like, it's about maintaining that brain, that grey brain matter, yeah. And basically keeping you active.' (participant 6)

Perceived physical benefits included impacts on the disease and symptoms *'until I was doing the exercise it was bad'* (participant 7), and *'I think my breathing is better'* (participant 1). And implications for physical function '*mobility, I think, it's one of the things that I have found so much better. Because when I joined there were lots of things I found really difficult*' (participant 1).

Perceived mood and cognition benefits were also noted. For example, improvements in confidence,

For peer review only - http://bmjopen.bmj.com/site/about/guidelines.xhtml

> 'You feel you can do more things. So, if there's an offer coming up for an outing to a garden all day, I know that I'm going to go' (participant 4)

And,

> 'Psychologically the illness has not taken over… because you're occupying your mind on something completely different, so you can't say ' oh I can't do that, I can't breathe', I can have a go. That's what life's all about at the end of the day' (participant 5)

Impacts on mood were driven by enjoying participation (theme 3) and a sense of the group's deep social cohesion (theme 4), see figure 1 (thematic map). Additionally, participants 1, 3 and 5 perceived these holistic health benefits as leading to reduced healthcare utilisation, *'I haven't had a bad bronchitis episode since I've been doing it'* (participant 1), and *'you're not so prone to picking up all these chest infections'* (participant 3).

Dance as an important part of their lives

Dance was perceived as an important part of their lives, *'It's just part of my life now'* (participant 3). For some participants dance had been important throughout their lives, while for others, dance only gained importance since joining the group *'I was your typical wallflower …. I never really got into dancing at all. Till now.'* (participant 3).

In general, the importance of dance participation was not related to their perceived ability - *'I wouldn't call myself twinkle toes'* (participant 3) and *'I can't dance for toffee, and I can't sing, but hey (laughs) who cares.'* (participant 1). However, perceptions regarding dance ability influenced how participants described what they do during the sessions. For example, some participants commented that they were not really dancing as they felt that a certain skill level was required for their individual definition of dance, instead describing the sessions as *'movement to music'* (participant 2). Nevertheless, learning to dance was frequently stated as a component of the sessions, hence, differing definitions of dance likely represented differing life experiences of dance, rather than differing views on what they gained from participation in the group.

> 'I'm learning something new and it's also, you come in and learn a dance that six weeks ago you never thought you'd get your feet around.' (participant 6)

This satisfaction related to a sense of mastery was also important. Observation data also noted participants improved over the sessions, particularly completing routines with less errors, with a clear sense of achievement-related satisfaction, perhaps as a contrast to a background of decline in other aspects of their functioning. Structured observations also noted that participants were often quite elegantly dressed, which may not be expected in activities seen as purely exercise.

Dance as an enjoyable activity

All participants greatly enjoyed the sessions, consistently describing it as fun, which was a main driver of attendance. This was repeatedly emphasised as being of major importance, and something participants felt was not appreciated or considered in other therapeutic exercise they had attended. In this sense dancing was viewed as an end-in-itself, rather than the means to an end, as was the case for gym-based exercise training. Multiple factors contributed to their enjoyment including a light-hearted and non-judgemental atmosphere, in which participants felt active contributors rather than passive recipients of care.

For peer review only - http://bmjopen.bmj.com/site/about/guidelines.xhtml

> 'There is this immense feeling of contentment, and it’s great. You know, when you've made an effort, you've done it, and jolly good. And that’s it.' (participant 2)

The atmosphere in the sessions was very relaxed. The element of humour was highlighted in structured observations, with regular group laughter noted, with a very inclusive quality.

> ‘…before you know it, we’re all in stiches. You know it’s something, you know. Its promoted in the group. You can feel it. You get the buzz. You have a good laugh and, apart from doing your dancing you’re having fun, even with the dancing you’re having fun.’ (participant 3)

Not feeling intimidated or judged was key to the enjoyment and contrasted with other exercise modalities and settings

> 'I won't go to the gym where there’s a lot of younger people than myself. Because they can be intimidating. So, I won’t do nothing like that.’ (participant 5)

Dance as a source of deep social cohesion

Participants described the other group members in very affectionate terms – *'I love everyone I'm with', 'we're like a small knit family'* (participant 6), and greatly valued their relationships with other individuals and the group as a whole.

> 'It’s not just about dancing it’s about the whole, being part of the organisation, being part of a little unit that I think I enjoy coming out.' (participant 6)

Having shared experience was extremely important to participants. Participants commented on feeling ‘other’ when in public or with friends outside of the group. Paradoxically, although the group is built around having respiratory disease, not feeling ‘other’ created an environment where the disease could be forgotten, providing a form of respite.

> 'I do it for the social side as well. Because it’s very nice to meet people with the same basic difficulties' (participant 2)

> 'As a group, you know, we obviously bonded with each other, well we know each other, it is, you know it’s something that…. I think we all enjoy it.' (participant 3)

> 'we kind of understand each other when we can't breathe' (participant 6)

> 'But I think that for me it’s about forgetting what’s wrong with me until I actually go home.' (participant 6)

The depth of connection between participants underpinned the holistic impacts described in theme 1. The shared experience enabled participants to develop a combination of acceptance and increased motivation for improvement. This forged a collective self-motivation, with participants seeing their health as their own responsibility, directly related to the effort they put in *'you got to be up for it, you've got to be disciplined'* (participant 4). Yet, simultaneously appreciating that this could be best achieved together.

> 'for me it’s a good thing. It's basically being with likeminded people, people who understand how you feel. You can see them, you can encourage them to do a little better. I think I've actually changed my attitude. Instead of being in denial about not being able to breathe, I think I've more embraced it.' (participant 6)

For peer review only - http://bmjopen.bmj.com/site/about/guidelines.xhtml

This deep social cohesion was attributed to the content of the sessions, particularly the presence of music, and dancing together.

> 'Well it means you accept the whole thing. You're moving with a group of people who have a similar problem.' (participant 2)

The supportive atmosphere was also attributed to session leader SW. SW, is very supportive while challenging the participants to do the best they can, which is greatly appreciated by the participants who describe her as 'a lovely person'. There is a clear focus on abilities rather than impairment, which helps set the tone for the entire group.

> '...the wonderful thing is, nobody's ever been told "you're useless and you can't do it' (participant 1)

> (referring to the group leader's responses) 'It's the effort that one puts into it, and that is repaid enormously' (participant 2)

The social relationships that have developed were also contrasted with other types of therapeutic exercise such as going to the gym, where they felt this depth of relationship was not created.

> 'if you go to the gym, you're just doing your own thing, whereas here we are supporting each other' (participant 1)

Discussion

This study found that long-standing participants in a dance group for people with CRD perceived a range of holistic health and wellbeing benefits. The dance group had become an important part of their lives. Enjoyment of participation and the development of deep social cohesion between participants were considered key drivers of these impacts.

This study has multiple strengths. To our knowledge it is the first study to explore community dance participation for individuals with respiratory disease in a real-world setting. A recent pilot study included qualitative aspects, based on informal group discussions and field notes (31), but no other studies have used in-depth qualitative interviews with long-term attendees, out-side of a trial setting, which improves the relevance of our work to real-world experience. Indeed, few qualitative studies of arts-in-health interventions in respiratory disease have explored such long-term participants' experiences. Secondly, the use of semi-structured interviews enabled inductive exploration of specific topics of interest. Thirdly, interpretation of the interview data was strengthened though use of structured observations.

Some limitations should be noted. Firstly, sample size was limited by the number of group participants. However, it was decided that sufficient data were collected to justify the themes. Secondly, as we only focused on a single group, the transferability of findings cannot be ascertained. Thirdly, more detailed clinical characterisation of the participants' respiratory disease may have been of interest. However, we feel it was adequate for our research topic and findings. Fourthly, a degree of selection bias may exist, as study participants were individuals who had maintained attendance, while those that did not enjoy the group no longer participated and were not included in the research.

Our findings echo some of the findings from the limited existing research regarding dance for people with CRD. A recent study assessing a dance intervention for COPD showed it to be feasible, with secondary outcome measures suggesting improvements in functional capacity, balance, anxiety and depression, physical activity, and health-related quality of life (32). These findings suggest that

For peer review only - http://bmjopen.bmj.com/site/about/guidelines.xhtml

elements of the perceived holistic benefits described in our study have quantifiable impacts using established assessment tools. Additionally, a second feasibility study assessing dance for people with breathlessness reported preliminary results suggestive dancing together is of importance, and participants reported 'coming alive' (31). Such findings appear similar to themes three and four of our study. The themes identified here also map to multiple levels of Maslow's hierarchy of needs model of human motivation (34) including safety, love and belonging, self-esteem, and self-actualisation. This supports our interpretation, that their experience as being complex and holistically beneficial, drove their motivation for participation. Overall, our findings are broadly corroborative with existing studies, while deepening understanding of underlying drivers for participation and psychological benefits observed.

Research exploring the experiences of people with CRD participating in other related activities shows some similarities and differences. As with the current findings, group support and increased self-confidence have been found to promote adherence to pulmonary rehabilitation (35). However, lack of social support and overcoming the effort of living with COPD, as well as anxiety and depression (8) have been identified as negatively influencing participation (35). The participants in our study showed consistent attendance over an extended period, despite often living alone or reporting limited social contacts. Potential explanations include the dance group providing an experience sufficiently different from PR to overcome these issues, with participants reporting strong social connectiveness with peers, and high levels of confidence in their abilities, despite the evident disease-based limitations. A related activity is SLH which consists of specially developed group singing sessions for people with CRD. Research on SLH has found very similar impacts to the current study, including positive impacts on general wellbeing, community/social support and enjoyment of participation. Both suggest physical benefits (13-15). Our findings echo those of research on community dance interventions in other patient groups including improving physical performance, mood, cognition, quality of life (26, 36, 37) sense of joy and pride (38), interpersonal relationships and connections (39). Hence, the perceived health impacts here reported may not be disease specific, but rather represent the impact of group dance participation on people in general. That said, given the biopsychosocial impacts of CRD and relative lack of effective management options for psychosocial components, dance interventions appear particularly well suited for people with CRDs.

Conclusion

CRDs are common and result in a high burden of morbidity, mortality and related economic implications. Therefore, low-cost, low-resource interventions with holistic impacts on health and wellbeing should be of broad interest. In particular, the perceived impacts on physical performance, psychosocial health, and health resource utilisation are promising.

Participants perceived a broad range of positive holistic health and wellbeing benefits; viewed participation as an important part of their lives; greatly enjoyed participation; and felt deep social cohesion with other participants. Further research is required to confirm, clarify and quantify these impacts.

Information on lead researcher:

Dr Keir Philip (KP) is a male, 33-year-old, respiratory physician who previously worked as a dancer and dance teacher, with experience leading community dance and dance for people with respiratory

For peer review only - http://bmjopen.bmj.com/site/about/guidelines.xhtml

disease. KP has received training in qualitative research methods from the Imperial College, University College London and through self-directed learning. He is currently completing a PhD at Imperial College, using both qualitative and quantitative methods. Qualifications: MBChB BSc MRCP EADTMH DPMSA.

KP and AL are dancers recreationally and previous dance experiences enabled discussions throughout the analysis to be explored fully and assumptions challenged within the researchers' assumptions of what dance is.

Acknowledgements

We would like to give our thanks to the study participants for their time and effort.

For peer review only

References

1. Ferkol T, Schraufnagel D. The global burden of respiratory disease. Annals of the American Thoracic Society. 2014;11(3):404-6.
2. Kelly JL, Bamsey O, Smith C, Lord VM, Shrikrishna D, Jones PW, et al. Health status assessment in routine clinical practice: the chronic obstructive pulmonary disease assessment test score in outpatients. Respiration. 2012;84(3):193-9.
3. Gardiner C, Gott M, Payne S, Small N, Barnes S, Halpin D, et al. Exploring the care needs of patients with advanced COPD: an overview of the literature. Respir Med. 2010;104(2):159-65.
4. Yohannes AM, Alexopoulos GS. Depression and anxiety in patients with COPD. Eur Respir Rev. 2014;23(133):345-9.
5. McCarthy B, Casey D, Devane D, Murphy K, Murphy E, Lacasse Y. Pulmonary rehabilitation for chronic obstructive pulmonary disease. The Cochrane database of systematic reviews. 2015(2):CD003793.
6. Hopkinson NS, Molyneux A, Pink J, Harrisingh MC. Chronic obstructive pulmonary disease: diagnosis and management: summary of updated NICE guidance. BMJ. 2019;366:l4486.
7. Rochester CL, Vogiatzis I, Holland AE, Lareau SC, Marciniuk DD, Puhan MA, et al. An Official American Thoracic Society/European Respiratory Society Policy Statement: Enhancing Implementation, Use, and Delivery of Pulmonary Rehabilitation. American journal of respiratory and critical care medicine. 2015;192(11):1373-86.
8. Boutou AK, Tanner RJ, Lord VM, Hogg L, Nolan J, Jefford H, et al. An evaluation of factors associated with completion and benefit from pulmonary rehabilitation in COPD. BMJ Open Respir Res. 2014;1(1):e000051.
9. Polkey MI, Qiu Z-H, Zhou L, Zhu M-D, Wu Y-X, Chen Y-Y, et al. Tai Chi and Pulmonary Rehabilitation Compared for Treatment-Naive Patients With COPD: A Randomized Controlled Trial. Chest. 2018;153(5):1116-24.
10. Lewis A, Hopkinson NS. Tai Chi Movements for Wellbeing – evaluation of a British Lung Foundation pilot. Perspectives in Public Health. 2019:1757913919872515.
11. Nolan CM, Rochester CL. Exercise Training Modalities for People with Chronic Obstructive Pulmonary Disease. Copd. 2019;16(5-6):378-89.
12. Lewis A, Cave P, Hopkinson NS. Singing for Lung Health: a qualitative assessment of a British Lung Foundation programme for group leaders. BMJ open respiratory research. 2017;4(1):e000216.
13. Lewis A, Cave P, Hopkinson NS. Singing for Lung Health: service evaluation of the British Lung Foundation programme. Perspect Public Health. 2018:1757913918774079.
14. Lewis A, Cave P, Stern M, Welch L, Taylor K, Russell J, et al. Singing for Lung Health-a systematic review of the literature and consensus statement. NPJ primary care respiratory medicine. 2016;26:16080.
15. Lord VM, Cave P, Hume VJ, Flude EJ, Evans A, Kelly JL, et al. Singing teaching as a therapy for chronic respiratory disease--a randomised controlled trial and qualitative evaluation. BMC pulmonary medicine. 2010;10:41.
16. Lord VM, Hume VJ, Kelly JL, Cave P, Silver J, Waldman M, et al. Singing classes for chronic obstructive pulmonary disease: a randomized controlled trial. BMC pulmonary medicine. 2012;12:69.
17. Drinkwater C, Wildman J, Moffatt S. Social prescribing. Bmj. 2019;364:l1285.
18. Fancourt D, Finn S. What is the evidence on the role of the arts in improving health and well-being? A scoping review. World Health Organisation; 2019.
19. Fancourt D, Steptoe A. The art of life and death: 14 year follow-up analyses of associations between arts engagement and mortality in the English Longitudinal Study of Ageing. BMJ. 2019;367:l6377.
20. Philip K, Lewis A, Hopkinson NS. Music and dance in chronic lung disease. Breathe. 2019;15(2):116-20.

For peer review only - http://bmjopen.bmj.com/site/about/guidelines.xhtml

21. Fong Yan A, Cobley S, Chan C, Pappas E, Nicholson LL, Ward RE, et al. The Effectiveness of Dance Interventions on Physical Health Outcomes Compared to Other Forms of Physical Activity: A Systematic Review and Meta-Analysis. Sports Med. 2018;48(4):933-51.
22. Hwang PW, Braun KL. The Effectiveness of Dance Interventions to Improve Older Adults' Health: A Systematic Literature Review. Altern Ther Health Med. 2015;21(5):64-70.
23. Jeong YJ, Hong SC, Lee MS, Park MC, Kim YK, Suh CM. Dance movement therapy improves emotional responses and modulates neurohormones in adolescents with mild depression. Int J Neurosci. 2005;115(12):1711-20.
24. Merom D, Ding D, Stamatakis E. Dancing Participation and Cardiovascular Disease Mortality: A Pooled Analysis of 11 Population-Based British Cohorts. American journal of preventive medicine. 2016;50(6):756-60.
25. Murrock CJ, Graor CH. Effects of dance on depression, physical function, and disability in underserved adults. J Aging Phys Act. 2014;22(3):380-5.
26. Patterson KK, Wong JS, Prout EC, Brooks D. Dance for the rehabilitation of balance and gait in adults with neurological conditions other than Parkinson's disease: A systematic review. Heliyon. 2018;4(3):e00584.
27. Rodrigues-Krause J, Krause M, Reischak-Oliveira A. Dancing for Healthy Aging: Functional and Metabolic Perspectives. Altern Ther Health Med. 2019;25(1):44-63.
28. Sharp K, Hewitt J. Dance as an intervention for people with Parkinson's disease: a systematic review and meta-analysis. Neurosci Biobehav Rev. 2014;47:445-56.
29. Philip KEJ. Sing a song of lung health-beyond the UK. Bmj. 2019;364:l652.
30. Philip KE, Akylbekov A, Stambaeva B, Sooronbaev T, Jones R. Music, Dance, and Harmonicas for People With COPD. Respiratory care. 2019;64(3):359.
31. Harrison SL BK, Edwards J, McFaull V, McLusky S, Russell A, Williams G, Williams S. DANCE FOR PEOPLE WITH CHRONIC BREATHLESSNESS: A FEASIBILITY STUDY. Thorax2019.
32. Wshah A, Butler S, Patterson K, Goldstein R, Brooks D. "Let's Boogie": FEASIBILITY OF A DANCE INTERVENTION IN PATIENTS WITH CHRONIC OBSTRUCTIVE PULMONARY DISEASE. J Cardiopulm Rehabil Prev. 2019;39(5):E14-E9.
33. Braun VC, V.,. Using thematic analysis in psychology. Qualitative Research in Psychology. 2006;3(2):77-101.
34. Maslow AH. A theory of human motivation. Psychology Review. 1943;50(4):370–96.
35. Arnold E, Bruton A, Ellis-Hill C. Adherence to pulmonary rehabilitation: A qualitative study. Respir Med. 2006;100(10):1716-23.
36. Eyigor S, Karapolat H, Durmaz B, Ibisoglu U, Cakir S. A randomized controlled trial of Turkish folklore dance on the physical performance, balance, depression and quality of life in older women. Arch Gerontol Geriatr. 2009;48(1):84-8.
37. Granacher U, Muehlbauer T, Bridenbaugh SA, Wolf M, Roth R, Gschwind Y, et al. Effects of a salsa dance training on balance and strength performance in older adults. Gerontology. 2012;58(4):305-12.
38. Alfredsson Olsson E, Heikkinen S. "I will never quit dancing". The emotional experiences of social dancing among older persons. J Aging Stud. 2019;51:100786.
39. Atkins R, Deatrick JA, Gage GS, Earley S, Earley D, Lipman TH. Partnerships to Evaluate the Social Impact of Dance for Health: A Qualitative Inquiry. J Community Health Nurs. 2019;36(3):124-38.

For peer review only

Thematic Map

A thematic map showing the main four themes and the keyways they relate to one another.

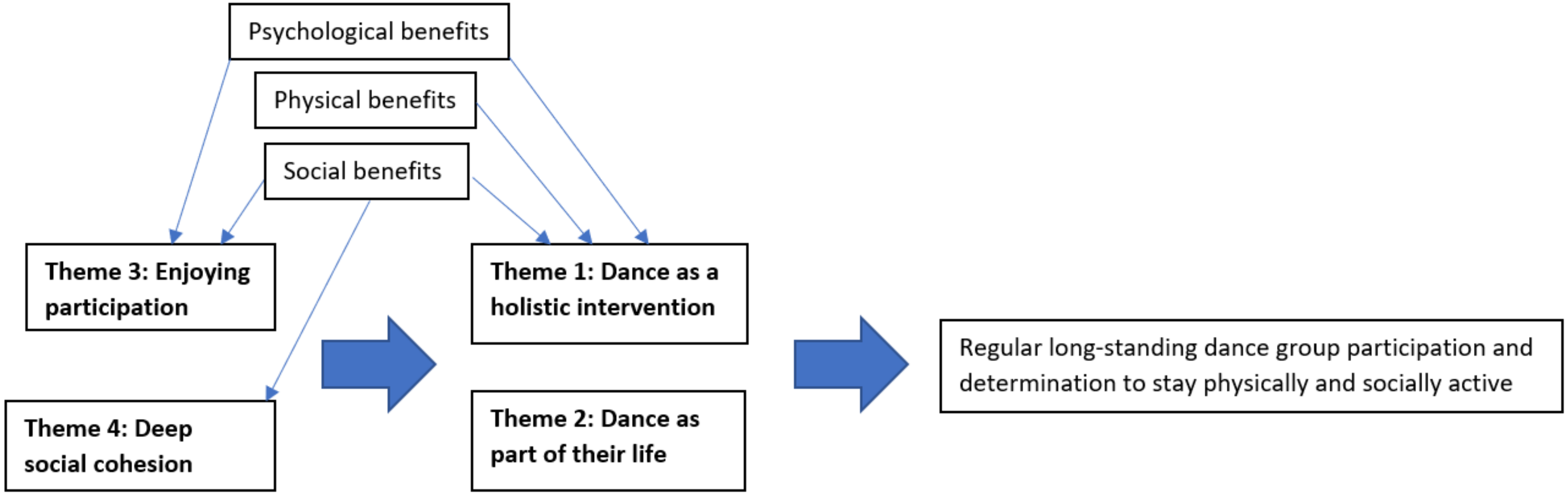

Title of Project:

Activity, Balance and Chronic Obstructive Pulmonary Disease (ABCOPD) study: Qualitative Dance Component

IRAS Project ID: 258399

Topic Guide for Interviews

Topics listed below were trialled and refined with respiratory specialist patient advisory groups prior to formal data collection.

All recordings should start with a clear statement of study participation consent in addition to the written. Initially discussion should start with rapport building with study participants. All interviews and focus groups will have audio recording on a dictaphone.

Key Topics:

How does having [primary respiratory diagnosis] affect your life?

- Symptoms
- Mood/mental health
- Socially

Physical activity (how much people do) in general life:

- What do you know about physical activity?
- What does it mean to you?
- How physically active are you?
- Has your experience of physical activity changed over time?
  -and living with lung disease?
- Should activity be avoided or actively engaged in?
- What sort of things can be done to improve physical activity levels in general?
  -and for you?

Physical capacity and physical performance (how much people can do and how well they do it)

- How would you describe the quality of your movement (in relation to stability, speed, strength and coordination?)
- Has the quality of your movement changed?
- Has your experience of your movement quality changed, for example do you find balancing, moving, or doing daily tasks more or less challenging than previously?

Experience of dance participation

How long have you been dancing?
Why do you do it?
Tell me about the dance group
How do you feel during the dance sessions?
What do you enjoy?
What do you not enjoy?

Impact of participation

What do you think are the main impacts of participating in the dance group?
Main positives?
Main negatives?
How has dancing impacted on your experience of living with lung disease?
Do you have any ideas for improving the classes?
What would you tell others who have lung disease about dancing?

Any other comments

13-FEB-19 V1

For peer review only

COREQ (COnsolidated criteria for REporting Qualitative research) Checklist

A checklist of items that should be included in reports of qualitative research. You must report the page number in your manuscript where you consider each of the items listed in this checklist. If you have not included this information, either revise your manuscript accordingly before submitting or note N/A.

Topic	Item No.	Guide Questions/Description	Reported on Page No.
**Domain 1: Research team and reflexivity**			
Personal characteristics			
Interviewer/facilitator	1	Which author/s conducted the interview or focus group?	
Credentials	2	What were the researcher's credentials? E.g. PhD, MD	
Occupation	3	What was their occupation at the time of the study?	
Gender	4	Was the researcher male or female?	
Experience and training	5	What experience or training did the researcher have?	
Relationship with participants			
Relationship established	6	Was a relationship established prior to study commencement?	
Participant knowledge of the interviewer	7	What did the participants know about the researcher? e.g. personal goals, reasons for doing the research	
Interviewer characteristics	8	What characteristics were reported about the inter viewer/facilitator? e.g. Bias, assumptions, reasons and interests in the research topic	
**Domain 2: Study design**			
Theoretical framework			
Methodological orientation and Theory	9	What methodological orientation was stated to underpin the study? e.g. grounded theory, discourse analysis, ethnography, phenomenology, content analysis	
Participant selection			
Sampling	10	How were participants selected? e.g. purposive, convenience, consecutive, snowball	
Method of approach	11	How were participants approached? e.g. face-to-face, telephone, mail, email	
Sample size	12	How many participants were in the study?	
Non-participation	13	How many people refused to participate or dropped out? Reasons?	
Setting			
Setting of data collection	14	Where was the data collected? e.g. home, clinic, workplace	
Presence of non-participants	15	Was anyone else present besides the participants and researchers?	
Description of sample	16	What are the important characteristics of the sample? e.g. demographic data, date	
Data collection			
Interview guide	17	Were questions, prompts, guides provided by the authors? Was it pilot tested?	
Repeat interviews	18	Were repeat inter views carried out? If yes, how many?	
Audio/visual recording	19	Did the research use audio or visual recording to collect the data?	
Field notes	20	Were field notes made during and/or after the inter view or focus group?	
Duration	21	What was the duration of the inter views or focus group?	
Data saturation	22	Was data saturation discussed?	
Transcripts returned	23	Were transcripts returned to participants for comment and/or	

For peer review only

Topic	Item No.	Guide Questions/Description	Reported on Page No.
		correction?	
**Domain 3: analysis and findings**			
Data analysis			
Number of data coders	24	How many data coders coded the data?	
Description of the coding tree	25	Did authors provide a description of the coding tree?	
Derivation of themes	26	Were themes identified in advance or derived from the data?	
Software	27	What software, if applicable, was used to manage the data?	
Participant checking	28	Did participants provide feedback on the findings?	
Reporting			
Quotations presented	29	Were participant quotations presented to illustrate the themes/findings? Was each quotation identified? e.g. participant number	
Data and findings consistent	30	Was there consistency between the data presented and the findings?	
Clarity of major themes	31	Were major themes clearly presented in the findings?	
Clarity of minor themes	32	Is there a description of diverse cases or discussion of minor themes?	

Developed from: Tong A, Sainsbury P, Craig J. Consolidated criteria for reporting qualitative research (COREQ): a 32-item checklist for interviews and focus groups. *International Journal for Quality in Health Care*. 2007. Volume 19, Number 6: pp. 349 – 357

Once you have completed this checklist, please save a copy and upload it as part of your submission. DO NOT include this checklist as part of the main manuscript document. It must be uploaded as a separate file.

For peer review only

Dance for people with chronic respiratory disease: A qualitative study

Journal:	*BMJ Open*
Manuscript ID	bmjopen-2020-038719.R1
Article Type:	Original research
Date Submitted by the Author:	11-Jul-2020
Complete List of Authors:	Philip, Keir; National Heart and Lung Institute,<br>Lewis, Adam; Brunel University London<br>Williams, Sian; National Heart and Lung Institute<br>Buttery, Sara; National Heart and Lung Institute<br>Polkey, Mike; Royal Bromton and Harefield NHS Trust,<br>Man, William; Royal Brompton & Harefield, Respiratory Medicine<br>Fancourt, Daisy; University College London Research Department of Epidemiology and Public Health, Department of Behavioural Science and Health<br>Hopkinson, Nicholas; Imperial College London, National Heart and Lung Institute
**Primary Subject Heading**:	Respiratory medicine
Secondary Subject Heading:	Complementary medicine, Patient-centred medicine, Qualitative research, Rehabilitation medicine, Sports and exercise medicine
Keywords:	Adult thoracic medicine < THORACIC MEDICINE, Chronic airways disease < THORACIC MEDICINE, REHABILITATION MEDICINE, COMPLEMENTARY MEDICINE

SCHOLARONE™ Manuscripts

I, the Submitting Author has the right to grant and does grant on behalf of all authors of the Work (as defined in the below author licence), an exclusive licence and/or a non-exclusive licence for contributions from authors who are: i) UK Crown employees; ii) where BMJ has agreed a CC-BY licence shall apply, and/or iii) in accordance with the terms applicable for US Federal Government officers or employees acting as part of their official duties; on a worldwide, perpetual, irrevocable, royalty-free basis to BMJ Publishing Group Ltd ("BMJ") its licensees and where the relevant Journal is co-owned by BMJ to the co-owners of the Journal, to publish the Work in this journal and any other BMJ products and to exploit all rights, as set out in our licence.

The Submitting Author accepts and understands that any supply made under these terms is made by BMJ to the Submitting Author unless you are acting as an employee on behalf of your employer or a postgraduate student of an affiliated institution which is paying any applicable article publishing charge ("APC") for Open Access articles. Where the Submitting Author wishes to make the Work available on an Open Access basis (and intends to pay the relevant APC), the terms of reuse of such Open Access shall be governed by a Creative Commons licence – details of these licences and which Creative Commons licence will apply to this Work are set out in our licence referred to above.

Other than as permitted in any relevant BMJ Author's Self Archiving Policies, I confirm this Work has not been accepted for publication elsewhere, is not being considered for publication elsewhere and does not duplicate material already published. I confirm all authors consent to publication of this Work and authorise the granting of this licence.

For peer review only

Dance for people with chronic respiratory disease: A qualitative study

Authors: Keir EJ Philip[1] *, Adam Lewis[2], Siân Williams[1], Sara C Buttery[1], Michael I Polkey[1,3], William D-C Man[3], Daisy Fancourt[4], Nicholas S Hopkinson[1]

Affiliations:

1) National Heart and Lung Institute, Imperial College London
2) Brunel University London
3) Respiratory Medicine, Royal Brompton and Harefield NHS Foundation Trust
4) Department of Behavioural Science and Health, University College London

*Corresponding author

Author Email Addresses

- Keir Philip: k.philip@imperial.ac.uk
- Adam Lewis: Adam.Lewis@brunel.ac.uk
- Siân Williams: sian.health@gmail.com
- Sara Buttery: s.buttery@rbht.nhs.uk
- Michael Polkey: m.polkey@rbht.nhs.uk
- William Man: w.man@rbht.nhs.uk
- Daisy Fancourt: d.fancourt@ucl.ac.uk
- Nicholas Hopkinson: n.hopkinson@imperial.ac.uk

Key Words: respiratory, dance, breathlessness, physical activity, rehabilitation.

Manuscript word count: 4176 (including quotes)

For peer review only - http://bmjopen.bmj.com/site/about/guidelines.xhtml

Abstract

Objectives: To explore the experiences and perceived impact on health and well-being related to participation in a dance group for people with chronic respiratory disease (CRD).

Design: An exploratory qualitative study using thematic analysis of semi-structured interviews.

Setting: A community dance group in a UK health centre.

Participants: Convenience sample of long-term dance group participants.

Intervention: Weekly community dance sessions designed for people with breathlessness, lasting 75 minutes, led by a trained community dance leader.

Results: Convenience sample of eight participants, 6 females, aged 57 to 87 years (mean 75), with a median 2-year attendance at weekly dance sessions. Long-term attendance was driven by strongly held beliefs regarding the health and well-being benefits of participation. Four key themes were identified: Dance as (1) a holistically beneficial activity, with physical and psychosocial health benefits including improved or maintained physical fitness and psychological well-being, and reduced need for healthcare; (2) An integral part of their life; (3) An enjoyable activity; and (4) a source of deep social cohesion.

Conclusions: Dance group participants perceived a broad range of health benefits of relevance to the biopsychosocial impacts of their respiratory disease. The themes identified are useful in the ongoing planning and evaluation of dance as a holistic complex intervention for people with CRD. Further research is required to assess the extent of health impacts identified, and how dance might be most effectively placed as an option in the management of CRD.

Registered as a component of the ABCOPD study at the NIH U.S. National Library of Medicine ClinicalTrials.gov: NCT04006015

Article Summary: Strengths and Limitations of this study

- The first study of long-term real-world participants of a community dance group for people with chronic respiratory disease and breathlessness.
- In depth exploration of participant experience.
- The sample was from a single group which may limit transferability.
- Semi-structured interviews facilitated a depth of understanding of clinically relevant topics, aiding the potential application of findings

Original Protocol/topic guide

See attached (supplementary file)

For peer review only

Funding statement

KP was supported by the Imperial College Clinician Investigator Scholarship. DF was supported by the Wellcome Trust [205407/Z/16/Z]. The funders had no say in the design and conduct of the study; collection, management, analysis, and interpretation of the data; preparation, review, or approval of the manuscript; and decision to submit the manuscript for publication.

Competing Interests

Siân Williams is the founder of the dance group and leads the sessions. She was not involved in the thematic analysis.

No other conflicts of interest declared.

Authors Contributions

KP had the original idea for the study, led the study including design and ethical approval, and conducted and transcribed the interviews. KP and AL conducted the analysis and wrote the first draft of the manuscript. SW helped develop the study concept and focus, the content of the topic guide, and reviewed and suggested improvements to manuscript drafts. SB, MP, WM, DF and NH provided valuable input in developing the methodological approach, developing the protocol, gaining ethical approval. All authors (KP, AL, SW, SB, MP, WM, DF, NH) contributed to the study design, writing, reviewing and editing the manuscript, and approved the final manuscript for submission.

Reporting Checklist

See attached COREQ checklist.

Data sharing statement

Data from this study are not being made available for sharing as it would not be possible to anonymise the interview transcripts given the personal content included and the very limited number of Dance groups for respiratory conditions in London.

Patient and Public Involvement

Patients and public were not involved in the design of the study, however the study specifically focuses on patient/participant perceptions.

For peer review only

For peer review only - http://bmjopen.bmj.com/site/about/guidelines.xhtml

Introduction

Chronic respiratory diseases (CRD) are leading causes of global morbidity and mortality (1), often causing persistent and disabling symptoms including breathlessness, cough, skeletal muscle impairment and exercise limitation (2, 3). Additionally, psychosocial impacts such as depression, loneliness and social isolation are common (4, 5) and are associated with worse clinical outcomes such as hospital admission for respiratory disease (6). Physical activity, exercise training and pulmonary rehabilitation (PR) are core components of CRD management (7, 8). However, engagement is inhibited by symptoms, availability, accessibility and attractiveness of local options (9, 10). Recently, interest in alternative forms of physical activity and exercise training has increased including Tai Chi (11, 12), Yoga (13) and Singing for Lung Health (SLH) (14-18). Furthermore, the current focus on social prescribing means such approaches are of increasing interest (19). Though an extensive body of research suggests arts interventions can improve health and wellbeing (20, 21), respiratory specific arts-in-health research is currently limited. Nevertheless, evidence which exists suggests a range of biopsychosocial benefits (22, 23).

Dance has been shown to be beneficial in healthy people and individuals with a range of medical conditions. Impacts include improved physical performance, function, mood, and social engagement, which are all highly relevant for people with CRD (24-31). Furthermore, dance is a core aspect of societies and cultures globally, hence has great potential as an enjoyable, engaging intervention in respiratory care throughout the world (32, 33). Regarding dance for people with CRD, two feasibility studies found dance for CRD interventions to be feasible (34, 35), one also suggested dance may improve functional capacity, balance, anxiety and depression, physical activity, and health-related quality of life (35). However, no studies have assessed participants' experiences and perceptions regarding health and wellbeing impacts, which is important given the need to understand the potential of dance for wider application and how to implement and evaluate it in trials and clinical practice.

The objective of this study was to explore the experiences and perceived health and well-being impacts of participation in a community-based dance group for people with CRD - 'Dance Easy'.

Method

Research design

We performed an exploratory qualitative study using thematic analysis within a critical realist paradigm, to explore the perceptions of participants of a community dance group for people with CRD. The primary focus was their views on the impact of participation on their health and wellbeing.

We used semi-structured interviews (see topic guide appendix), developed by reviewing research on the impacts of conceptually related interventions such as PR, SLH and Tai-Chi for people with CRD. This contributed deductive elements to the analysis. However, analysis was primarily inductive using open-ended questions and open coding. Participants were encouraged to continue discussing topics which appeared meaningful with prompts. Interviews were complemented by structured observations by KP, who participated by dancing with the group over 4 sessions. This participation aimed to build rapport and trust with participants to facilitate interviews, and make structured observations to contextualise interview content, and triangulate themes. Additionally, this method

For peer review only - http://bmjopen.bmj.com/site/about/guidelines.xhtml

enabled greater reflexivity within the analysis. Participants were made aware that KP is a clinician (but not involved in their clinical care) with experience of community dance groups.

The eight regular group participants were approached face-to-face by the group leader, and all consented to participate (convenience sample) in the study. Eligibility criteria included a clinical diagnosis of CRD, age >18 years and able to consent. All participants received participant information sheets and provided signed informed consent. Ethical approval was granted by the Office for Research Ethics Committees Northern Ireland (ORECNI) (19/NI/0073).

Dance Group intervention

The group started in May 2017, with seven of the study participants present from the first few months. The group meets weekly in a community centre attached to a health centre in London. Participants usually meet in the café from 30 minutes before the session. The sessions are free for participants, the leader volunteers her time for free, and the space is provided by Whittington Health. The original participants were attendees at a 'Breathe Easy' support group for people with respiratory conditions, additional members became aware of the dance group through word of mouth.

Sessions last approximately 75 minutes and include a warmup, including qigong movements (see https://www.nqa.org/what-is-qigong- for more information on qigong), progressively complex group dance routines, warm-down, stretching and relaxation. The session leader (SW) is a trained community dance group leader with extensive experience working with people with CRD. Music is played throughout the sessions and includes an eclectic mix of genres, selected by the session leader and participants, to inspire dancing but not feel too fast (subjectively from the participant's perspective). Each track corresponds to a specific movement routine. Dances are selected from a repertoire, with new dances added gradually. Though led by SW, participants contribute their opinions, movements and artistic expressions.

The dances are sufficiently physically demanding to induce breathlessness, though not excessively, as judged by participants. Given the group's heterogeneity regarding disease severity, age, and co-morbidities, a degree of personalisation is required. This is achieved by providing optional variations on movements, and allowing participants to pace themselves and take breaks as required. An approach that sees the participant as the expert in their own condition and physical capacities, and trusts their ability to titrate their exertion accordingly. Sessions are structured yet relaxed, focusing on dance as an enjoyable activity. Short breaks between dances are included for recovery and are used creatively to talk about dance, music and other topics that emerge. After the dance session, four to six participants go to a local pub to eat and chat, which developed spontaneously and became an established routine.

Data collection

Convenience sampling was used. Interviews were conducted one-to-one, by KP (see researcher info). Reflections and impressions were documented immediately following the interviews, with memo writing continuing throughout the analytic process. Interviews were conducted privately in the room where the dance takes place, or the café, depending on participant preference. Interviews, recorded by dictaphone, lasted 24 to 43 minutes, and consisted of open-ended questions, semi-structured around the following topics:

- Experience of respiratory illness and impact on life
- Experience of dance group
- Perceived impacts of dance group participation

For peer review only - http://bmjopen.bmj.com/site/about/guidelines.xhtml

Data analysis

KP and AL conducted thematic analysis based on that outlined by Braun and Clarke (36) and Terry et al. (37). Interviews were transcribed verbatim by KP, then KP and AL conducted further analysis. During phase 1, transcripts were read and re-read, and audio recordings listened to repeatedly. In phase 2, open inclusive coding of transcripts was used. This generated first cycle codes, mainly in-vivo (verbatim), descriptive, process, emotive and value items. We chose this method to enable interpretive and more qualitative elements in our approach, which we felt was appropriate given the exploratory nature of this study. Second cycle coding used the context from the first cycle, then focused on discussions relating to dance, activity and their disease. Code mapping facilitated grouping of codes. Preliminary themes (phase 3) were constructed from code mapping and reorganisation in relation to the associated quotes. Phases 1-3 were completed by KP and AL independently, before coming together to alter, refine, consolidate and name themes (phase 4 and 5). Structured observations, reflexive accounts post-interview, and memos were incorporated to the thematic analysis. Themes were reviewed with the group leader SW. Participant respondent validation was not performed because of the value placed on researchers' interpretation within our methodology.

Demographic and disease specific information was sought. Breathlessness was to indicate severity, rather than lung function measures as breathlessness relates more closely to patient related outcomes including mortality and functional impairment (2). Furthermore, participants could provide this themselves, whereas hospital investigations were not readily accessible, and participants had different CRD, which limits the relevance of spirometry group averages to this study.

Results

Eight regular dance group participants were recruited, representing the group at that time, including 6 women and 2 men, aged 57-87 years (mean 75). All reported CRD, primary diagnoses were reported as COPD (x5), asthma (x2), bronchiectasis (x1), however multimorbidity was common including arthritis, diabetes, sarcoidosis, visual impairment, and haemochromotosis. Four participants had an MRC (Medical Research Council) breathlessness scores of 2, one had a score of 3, three had scores of 1. None used supplementary oxygen. Participants lived in houses (6) and flats (2). Half of the participants lived alone, the others co-habited with their partner or children. . Interviews lasted a mean of 29 minutes 46 seconds (range 23mins 47secs, to 43mins 14secs).

No adverse events were reported. Participants perceived a wide range of health and wellbeing benefits related to attendance. All were extremely positive about the group, none expressed significant negatives, though some experienced transport related challenges. Attendance was very good, both self-reported and according to the leader's register. Non-attendance was related to holidays, transport difficulties or health issues such as optician appointments. Participants showed marked determination regarding being physically active, including but not limited to dance (walking and gardening frequently mentioned), so that '*psychologically the illness has not taken over*' (participant 5). They perceived sedentarism as highly negative, often stating the only choice being to '*get on with it*' (participant 2). This determination and commitment was driven by the identified themes, which were dance as (1) A holistically beneficial activity; (2) An important part of their lives; (3) An enjoyable activity; and (4) A source of deep social cohesion. Themes 3 and 4 were drivers of themes 1 and 2, but also stood alone so were not considered subthemes.

Figure 1: Thematic Map

For peer review only - http://bmjopen.bmj.com/site/about/guidelines.xhtml

Dance as a holistically beneficial activity

Participants viewed the sessions as a combined exercise, dance, and social activity. They described physical, psychological and social benefits, which were synergistic and, largely inseparable from one another.

> ‘it’s a bit of everything, afternoon social activity, because everyone has tea when they finish, or pub. It’s a bit of exercise, a bit of, entertainment, it’s a bit of, overall, learning.’ (participant 6)

Participation was perceived as beneficial for overall health status - *‘I feel like it’s been a great benefit for me. It has impacted on me.’* (participant 3). Impacts were described as improvements *‘you’re able to do things now’* (participant 3), or physical and cognitive maintenance in the context of ageing:

> ‘…keeping one's body and one's mind active is very important. And that is really the be all of the end all [most important thing]. That’s the object of the exercise' (participant 2), ‘it helps you maintain a standard as it were, of operation in the world I suppose' (participant 2)

> And

> 'Also when the music comes on, you, about three beats and you're like, 'oh', so I like that because you're like, it’s about maintaining that brain, that grey brain matter, yeah. And basically keeping you active.’ (participant 6)

Perceived physical benefits included impacts on the disease and symptoms *‘until I was doing the exercise it was bad’* (participant 7), and *‘I think my breathing is better'* (participant 1). And implications for physical function '*mobility, I think, it’s one of the things that I have found so much better. Because when I joined there were lots of things I found really difficult*' (participant 1).

Perceived mood and cognition benefits were also noted. For example, improvements in confidence,

> 'You feel you can do more things. So, if there’s an offer coming up for an outing to a garden all day, I know that I'm going to go' (participant 4)

And,

> 'Psychologically the illness has not taken over… because you're occupying your mind on something completely different, so you can’t say ' oh I can't do that, I can't breathe', I can have a go. That's what life’s all about at the end of the day' (participant 5)

Impacts on mood were driven by enjoying participation (theme 3) and a sense of the group’s deep social cohesion (theme 4), see figure 1 (thematic map). Additionally, participants 1, 3 and 5 perceived these holistic health benefits as leading to reduced healthcare utilisation, *'I haven’t had a bad bronchitis episode since I’ve been doing it'* (participant 1), and ‘*you’re not so prone to picking up all these chest infections*’ (participant 3).

Dance as an important part of their lives

Dance was perceived as an important part of their lives, *‘It’s just part of my life now’* (participant 3). For some participants dance had been important throughout their lives (all but one reported dancing ‘socially’ at family events, 3 took classes), while for others, dance only gained importance since joining

For peer review only - http://bmjopen.bmj.com/site/about/guidelines.xhtml

the group *'I was your typical wallflower …. I never really got into dancing at all. Till now.'* (participant 3).

In general, the importance of dance participation was not related to their perceived ability or previous experience - *'I wouldn't call myself twinkle toes'* (participant 3) and *'I can't dance for toffee, and I can't sing, but hey (laughs) who cares.'* (participant 1). However, perceptions regarding dance ability influenced how participants described what they do during the sessions. For example, some participants commented that they were not really dancing as they felt that a certain skill level was required for their individual definition of dance, instead describing the sessions as '*movement to music'* (participant 2). Nevertheless, learning to dance was frequently stated as a component of the sessions, hence, differing definitions of dance likely represented differing life experiences of dance, rather than differing views on what they gained from participation in the group.

> 'I'm learning something new and it's also, you come in and learn a dance that six weeks ago you never thought you'd get your feet around.' (participant 6)

This satisfaction related to a sense of mastery was also important. Observation data also noted participants improved over the sessions, particularly completing routines with fewer errors, with a clear sense of achievement-related satisfaction, perhaps as a contrast to a background of decline in other aspects of their functioning. Structured observations also noted that participants were often quite elegantly dressed, which may not be expected in activities seen as purely exercise.

Dance as an enjoyable activity

All participants greatly enjoyed the sessions, consistently describing it as fun, which was a main driver of attendance. This was repeatedly emphasised as being of major importance, and something participants felt was not appreciated or considered in other therapeutic exercise they had attended. In this sense dancing was viewed as an end-in-itself, rather than the means to an end, as was the case for gym-based exercise training. Multiple factors contributed to their enjoyment including a light-hearted and non-judgemental atmosphere, in which participants felt active contributors rather than passive recipients of care.

> 'There is this immense feeling of contentment, and it's great. You know, when you've made an effort, you've done it, and jolly good. And that's it.' (participant 2)

The atmosphere in the sessions was very relaxed. The element of humour was highlighted in structured observations, with regular group laughter noted, with a very inclusive quality.

> '…before you know it, we're all in stiches. You know it's something, you know. Its promoted in the group. You can feel it. You get the buzz. You have a good laugh and, apart from doing your dancing you're having fun, even with the dancing you're having fun.' (participant 3)

Not feeling intimidated or judged was key to the enjoyment and contrasted with other exercise modalities and settings

> 'I won't go to the gym where there's a lot of younger people than myself. Because they can be intimidating. So, I won't do nothing like that.' (participant 5)

Dance as a source of deep social cohesion

Participants described the other group members in very affectionate terms – *'I love everyone I'm with', 'we're like a small knit family'* (participant 6), and greatly valued their relationships with other individuals and the group as a whole.

For peer review only - http://bmjopen.bmj.com/site/about/guidelines.xhtml

> 'It's not just about dancing it's about the whole, being part of the organisation, being part of a little unit that I think I enjoy coming out.' (participant 6)

Having shared experience was extremely important to participants. Participants commented on feeling 'other' when in public or with friends outside of the group. Paradoxically, although the group is built around having respiratory disease, not feeling 'other' created an environment where the disease could be forgotten, providing a form of respite.

> 'I do it for the social side as well. Because it's very nice to meet people with the same basic difficulties' (participant 2)

> 'As a group, you know, we obviously bonded with each other, well we know each other, it is, you know it's something that…. I think we all enjoy it.' (participant 3)

> 'we kind of understand each other when we can't breathe' (participant 6)

> 'But I think that for me it's about forgetting what's wrong with me until I actually go home.' (participant 6)

The depth of connection between participants underpinned the holistic impacts described in theme 1. The shared experience enabled participants to develop a combination of acceptance and increased motivation for improvement. This forged a collective self-motivation, with participants seeing their health as their own responsibility, directly related to the effort they put in *'you got to be up for it, you've got to be disciplined'* (participant 4). Yet, simultaneously appreciating that this could be best achieved together.

> 'for me it's a good thing. It's basically being with likeminded people, people who understand how you feel. You can see them, you can encourage them to do a little better. I think I've actually changed my attitude. Instead of being in denial about not being able to breathe, I think I've more embraced it.' (participant 6)

This deep social cohesion was attributed to the content of the sessions, particularly the presence of music, and dancing together.

> 'Well it means you accept the whole thing. You're moving with a group of people who have a similar problem.' (participant 2)

The supportive atmosphere was also attributed to session leader SW. SW, is very supportive while challenging the participants to do the best they can, which is greatly appreciated by the participants who describe her as 'a lovely person'. There is a clear focus on abilities rather than impairment, which helps set the tone for the entire group.

> '…the wonderful thing is, nobody's ever been told "you're useless and you can't do it' (participant 1)

> (referring to the group leader's responses) 'It's the effort that one puts into it, and that is repaid enormously' (participant 2)

The social relationships that have developed were also contrasted with other types of therapeutic exercise such as going to the gym, where they felt this depth of relationship was not created.

> 'if you go to the gym, you're just doing your own thing, whereas here we are supporting each other' (participant 1)

For peer review only - http://bmjopen.bmj.com/site/about/guidelines.xhtml

Discussion

This study found that long-standing participants in a dance group for people with CRD perceived a range of holistic health and wellbeing benefits. The dance group had become an important part of their lives. Enjoyment of participation and the development of deep social cohesion between participants were considered key drivers of these impacts.

This study has multiple strengths. To our knowledge it is the first study to explore community dance participation for individuals with respiratory disease in a real-world setting. A recent pilot study included qualitative aspects, based on informal group discussions and field notes (34), but no other studies have used in-depth qualitative interviews with long-term attendees, out-side of a trial setting, which improves the relevance of our work to real-world experience. Indeed, few qualitative studies of arts-in-health interventions in respiratory disease have explored such long-term participants' experiences. Secondly, the use of semi-structured interviews enabled inductive exploration of specific topics of interest. Thirdly, interpretation of the interview data was strengthened though use of structured observations.

Some limitations should be noted. Firstly, sample size was limited by the number of group participants. However, given the exploratory nature of the study, it was decided that sufficient data were collected to justify the themes. Secondly, as we only focused on a single group, the transferability of findings cannot be ascertained. Thirdly, more detailed clinical characterisation of the participants' respiratory disease may have been of interest to people reading this study if considering practical implications for their context. However, we collected the relevant data for our research question. Fourthly, a degree of selection bias may exist, as study participants were individuals who had maintained attendance, while those that did not enjoy the group no longer participated and were not included in the research. It would have been useful to interview people who no longer attended to explore the reasons for non-attendance. 'Similarly, the group has a small number of participants, which is likely due to no formal promotion activities because it is unfunded, and the queries are managed by the Breathe Easy team themselves. No formal referral pathway yet exists. It is possible the small group size may suggest that some potential participants did not see this as a desirable activity. However, given the lack of promotion activities it is not possible to say.

Our findings echo some of the findings from the limited existing research regarding dance for people with CRD. A recent study assessing a dance intervention for COPD showed it to be feasible, with secondary outcome measures suggesting improvements in functional capacity, balance, anxiety and depression, physical activity, and health-related quality of life (35). These findings suggest that elements of the perceived holistic benefits described in our study have quantifiable impacts using established assessment tools. Additionally, a second feasibility study assessing dance for people with breathlessness reported preliminary results suggestive dancing together is of importance, and participants reported 'coming alive' (34). Such findings appear similar to themes three and four of our study. The themes identified here also map to multiple levels of Maslow's hierarchy of needs model of human motivation (38) including safety, love and belonging, self-esteem, and self-actualisation. This supports our interpretation, that their experience as being complex and holistically beneficial, drove their motivation for participation. Overall, our findings are broadly corroborative with existing studies, while deepening understanding of underlying drivers for participation and psychological benefits observed.

Research exploring the experiences of people with CRD participating in related activities shows some similarities and differences. Regarding pulmonary rehabilitation (PR), a high-value intervention(39)

For peer review only - http://bmjopen.bmj.com/site/about/guidelines.xhtml

which commonly includes group based callisthenic type exercises, group support and increased self-confidence have been found to promote adherence (40). However, lack of social support and overcoming the effort of living with COPD, as well as anxiety and depression (10) have been identified as negatively influencing participation in PR (40). The participants in our study showed consistent attendance over an extended period, despite often living alone or reporting limited social contacts. Potential explanations include the dance group providing an experience sufficiently different from PR to overcome these issues, with participants reporting strong social connectiveness with peers, and high levels of confidence in their abilities, despite the evident disease-based limitations. A related activity is SLH which consists of specially developed group singing sessions for people with CRD. Research on SLH has found very similar impacts to the current study, including positive impacts on general wellbeing, community/social support and enjoyment of participation. Both suggest physical benefits (15-17). Our findings echo those of research on community dance interventions in different patient groups including improving physical performance, mood, cognition, quality of life (29, 41, 42) sense of joy and pride (43), interpersonal relationships and connections (44). Hence, the perceived health impacts here reported may not be disease specific, but rather represent the impact of group dance participation on people in general. That said, given the biopsychosocial impacts of CRD and relative lack of effective management options for psychosocial components, dance interventions appear particularly well suited for people with CRDs.

Of note, the study participants did not report severe breathlessness, none used supplementary oxygen, and all lived relatively independently. Given the strong evidence base for pulmonary rehabilitation and other forms of exercise in people with more severe breathlessness and disease related impairment, it would be of interest to explore the use of dance interventions in such individuals. Similarly, they were generally quite active people, who clearly valued various types of physical activity, this may frame their highly positive perspectives, and indicates further research on interventions targeting less-active individuals would be of value, as well as groups who meet via the internet.

Conclusion

CRDs are common and result in a high burden of morbidity, mortality and related economic implications. Therefore, low-cost, low-resource interventions with holistic impacts on health and wellbeing should be of broad interest. In particular, the perceived impacts on physical performance, psychosocial health, and health resource utilisation are promising. Furthermore, this study highlights the importance of developing enjoyable physical activities that provide opportunity for self-expression for people with CRD to utilise intrinsic motivation, as an end in itself, but also to support compliance.

Participants perceived a broad range of positive holistic health and wellbeing benefits; viewed participation as an important part of their lives; greatly enjoyed participation; and felt deep social cohesion with other participants. Further research is required to confirm, clarify and quantify these impacts, and assess the cost effectiveness of sustainable methods of delivery (i.e. paid session leaders and funding for space).

Information on lead researcher:

Dr Keir Philip (KP) is a male, 33-year-old, respiratory physician who previously worked as a dancer and dance teacher, with experience leading community dance and dance for people with respiratory

For peer review only - http://bmjopen.bmj.com/site/about/guidelines.xhtml

disease. KP has received training in qualitative research methods from the Imperial College, University College London and through self-directed learning. He is currently completing a PhD at Imperial College, using both qualitative and quantitative methods. Qualifications: MBChB BSc MRCP EADTMH DPMSA.

KP and AL are dancers recreationally and previous dance experiences enabled discussions throughout the analysis to be explored fully and assumptions challenged within the researchers' assumptions of what dance is.

Acknowledgements

We would like to give our thanks to the study participants for their time and effort.

For peer review only

For peer review only - http://bmjopen.bmj.com/site/about/guidelines.xhtml

References

1. Ferkol T, Schraufnagel D. The global burden of respiratory disease. Annals of the American Thoracic Society. 2014;11(3):404-6.
2. Kelly JL, Bamsey O, Smith C, Lord VM, Shrikrishna D, Jones PW, et al. Health status assessment in routine clinical practice: the chronic obstructive pulmonary disease assessment test score in outpatients. Respiration. 2012;84(3):193-9.
3. Nishiyama O, Taniguchi H, Kondoh Y, Kimura T, Ogawa T, Watanabe F, et al. Quadriceps weakness is related to exercise capacity in idiopathic pulmonary fibrosis. Chest. 2005;127(6):2028-33.
4. Gardiner C, Gott M, Payne S, Small N, Barnes S, Halpin D, et al. Exploring the care needs of patients with advanced COPD: an overview of the literature. Respir Med. 2010;104(2):159-65.
5. Yohannes AM, Alexopoulos GS. Depression and anxiety in patients with COPD. Eur Respir Rev. 2014;23(133):345-9.
6. Bu F, Philip K, Fancourt D. Social isolation and loneliness as risk factors for hospital admissions for respiratory disease among older adults. Thorax. 2020.
7. Spruit MA, Singh SJ, Garvey C, ZuWallack R, Nici L, Rochester C, et al. An official American Thoracic Society/European Respiratory Society statement: key concepts and advances in pulmonary rehabilitation. American journal of respiratory and critical care medicine. 2013;188(8):e13-64.
8. Hopkinson NS, Molyneux A, Pink J, Harrisingh MC. Chronic obstructive pulmonary disease: diagnosis and management: summary of updated NICE guidance. BMJ. 2019;366:l4486.
9. Rochester CL, Vogiatzis I, Holland AE, Lareau SC, Marciniuk DD, Puhan MA, et al. An Official American Thoracic Society/European Respiratory Society Policy Statement: Enhancing Implementation, Use, and Delivery of Pulmonary Rehabilitation. American journal of respiratory and critical care medicine. 2015;192(11):1373-86.
10. Boutou AK, Tanner RJ, Lord VM, Hogg L, Nolan J, Jefford H, et al. An evaluation of factors associated with completion and benefit from pulmonary rehabilitation in COPD. BMJ Open Respir Res. 2014;1(1):e000051.
11. Polkey MI, Qiu Z-H, Zhou L, Zhu M-D, Wu Y-X, Chen Y-Y, et al. Tai Chi and Pulmonary Rehabilitation Compared for Treatment-Naive Patients With COPD: A Randomized Controlled Trial. Chest. 2018;153(5):1116-24.
12. Lewis A, Hopkinson NS. Tai Chi Movements for Wellbeing – evaluation of a British Lung Foundation pilot. Perspectives in Public Health. 2019:1757913919872515.
13. Nolan CM, Rochester CL. Exercise Training Modalities for People with Chronic Obstructive Pulmonary Disease. Copd. 2019;16(5-6):378-89.
14. Lewis A, Cave P, Hopkinson NS. Singing for Lung Health: a qualitative assessment of a British Lung Foundation programme for group leaders. BMJ open respiratory research. 2017;4(1):e000216.
15. Lewis A, Cave P, Hopkinson NS. Singing for Lung Health: service evaluation of the British Lung Foundation programme. Perspect Public Health. 2018:1757913918774079.
16. Lewis A, Cave P, Stern M, Welch L, Taylor K, Russell J, et al. Singing for Lung Health-a systematic review of the literature and consensus statement. NPJ primary care respiratory medicine. 2016;26:16080.
17. Lord VM, Cave P, Hume VJ, Flude EJ, Evans A, Kelly JL, et al. Singing teaching as a therapy for chronic respiratory disease--a randomised controlled trial and qualitative evaluation. BMC pulmonary medicine. 2010;10:41.
18. Lord VM, Hume VJ, Kelly JL, Cave P, Silver J, Waldman M, et al. Singing classes for chronic obstructive pulmonary disease: a randomized controlled trial. BMC pulmonary medicine. 2012;12:69.
19. Drinkwater C, Wildman J, Moffatt S. Social prescribing. Bmj. 2019;364:l1285.
20. Fancourt D, Finn S. What is the evidence on the role of the arts in improving health and well-being? A scoping review. World Health Organisation; 2019.

For peer review only - http://bmjopen.bmj.com/site/about/guidelines.xhtml

21. Fancourt D, Steptoe A. The art of life and death: 14 year follow-up analyses of associations between arts engagement and mortality in the English Longitudinal Study of Ageing. BMJ. 2019;367:l6377.
22. Philip K, Lewis A, Hopkinson NS. Music and dance in chronic lung disease. Breathe. 2019;15(2):116-20.
23. Philip KEJ, Williams, S., Simpson, J., Rooney, C., Davey, C., Gupta, R., Singh, R.,. Exploring participants current experiences and opinions regarding use of music/dance in pulmonary rehabilitation. European Respiratory Society Conference; Milan, Italy. European Respiratory Journal2017.
24. Fong Yan A, Cobley S, Chan C, Pappas E, Nicholson LL, Ward RE, et al. The Effectiveness of Dance Interventions on Physical Health Outcomes Compared to Other Forms of Physical Activity: A Systematic Review and Meta-Analysis. Sports Med. 2018;48(4):933-51.
25. Hwang PW, Braun KL. The Effectiveness of Dance Interventions to Improve Older Adults' Health: A Systematic Literature Review. Altern Ther Health Med. 2015;21(5):64-70.
26. Jeong YJ, Hong SC, Lee MS, Park MC, Kim YK, Suh CM. Dance movement therapy improves emotional responses and modulates neurohormones in adolescents with mild depression. Int J Neurosci. 2005;115(12):1711-20.
27. Merom D, Ding D, Stamatakis E. Dancing Participation and Cardiovascular Disease Mortality: A Pooled Analysis of 11 Population-Based British Cohorts. American journal of preventive medicine. 2016;50(6):756-60.
28. Murrock CJ, Graor CH. Effects of dance on depression, physical function, and disability in underserved adults. J Aging Phys Act. 2014;22(3):380-5.
29. Patterson KK, Wong JS, Prout EC, Brooks D. Dance for the rehabilitation of balance and gait in adults with neurological conditions other than Parkinson's disease: A systematic review. Heliyon. 2018;4(3):e00584.
30. Rodrigues-Krause J, Krause M, Reischak-Oliveira A. Dancing for Healthy Aging: Functional and Metabolic Perspectives. Altern Ther Health Med. 2019;25(1):44-63.
31. Sharp K, Hewitt J. Dance as an intervention for people with Parkinson's disease: a systematic review and meta-analysis. Neurosci Biobehav Rev. 2014;47:445-56.
32. Philip KEJ. Sing a song of lung health-beyond the UK. Bmj. 2019;364:l652.
33. Philip KE, Akylbekov A, Stambaeva B, Sooronbaev T, Jones R. Music, Dance, and Harmonicas for People With COPD. Respiratory care. 2019;64(3):359.
34. Harrison SL BK, Edwards J, McFaull V, McLusky S, Russell A, Williams G, Williams S. DANCE FOR PEOPLE WITH CHRONIC BREATHLESSNESS: A FEASIBILITY STUDY. Thorax2019.
35. Wshah A, Butler S, Patterson K, Goldstein R, Brooks D. "Let's Boogie": FEASIBILITY OF A DANCE INTERVENTION IN PATIENTS WITH CHRONIC OBSTRUCTIVE PULMONARY DISEASE. J Cardiopulm Rehabil Prev. 2019;39(5):E14-E9.
36. Braun V, Clarke V.,. Using thematic analysis in psychology. Qualitative Research in Psychology. 2006;3(2):77-101.
37. Terry G, Hayfield, N., Clarke, V., Braun, V.,. Thematic Analysis in: The SAGE Handbook of Qualitative Research in Psychology. London: SAGE Publications Ltd; 2017.
38. Maslow AH. A theory of human motivation. Psychology Review. 1943;50(4):370–96.
39. Zoumot Z, Jordan S, Hopkinson NS. Emphysema: time to say farewell to therapeutic nihilism. Thorax. 2014;69(11):973-5.
40. Arnold E, Bruton A, Ellis-Hill C. Adherence to pulmonary rehabilitation: A qualitative study. Respir Med. 2006;100(10):1716-23.
41. Eyigor S, Karapolat H, Durmaz B, Ibisoglu U, Cakir S. A randomized controlled trial of Turkish folklore dance on the physical performance, balance, depression and quality of life in older women. Arch Gerontol Geriatr. 2009;48(1):84-8.

For peer review only - http://bmjopen.bmj.com/site/about/guidelines.xhtml

42. Granacher U, Muehlbauer T, Bridenbaugh SA, Wolf M, Roth R, Gschwind Y, et al. Effects of a salsa dance training on balance and strength performance in older adults. Gerontology. 2012;58(4):305-12.
43. Alfredsson Olsson E, Heikkinen S. "I will never quit dancing". The emotional experiences of social dancing among older persons. J Aging Stud. 2019;51:100786.
44. Atkins R, Deatrick JA, Gage GS, Earley S, Earley D, Lipman TH. Partnerships to Evaluate the Social Impact of Dance for Health: A Qualitative Inquiry. J Community Health Nurs. 2019;36(3):124-38.

For peer review only

Thematic Map

A thematic map showing the main four themes and the key ways they relate to one another.

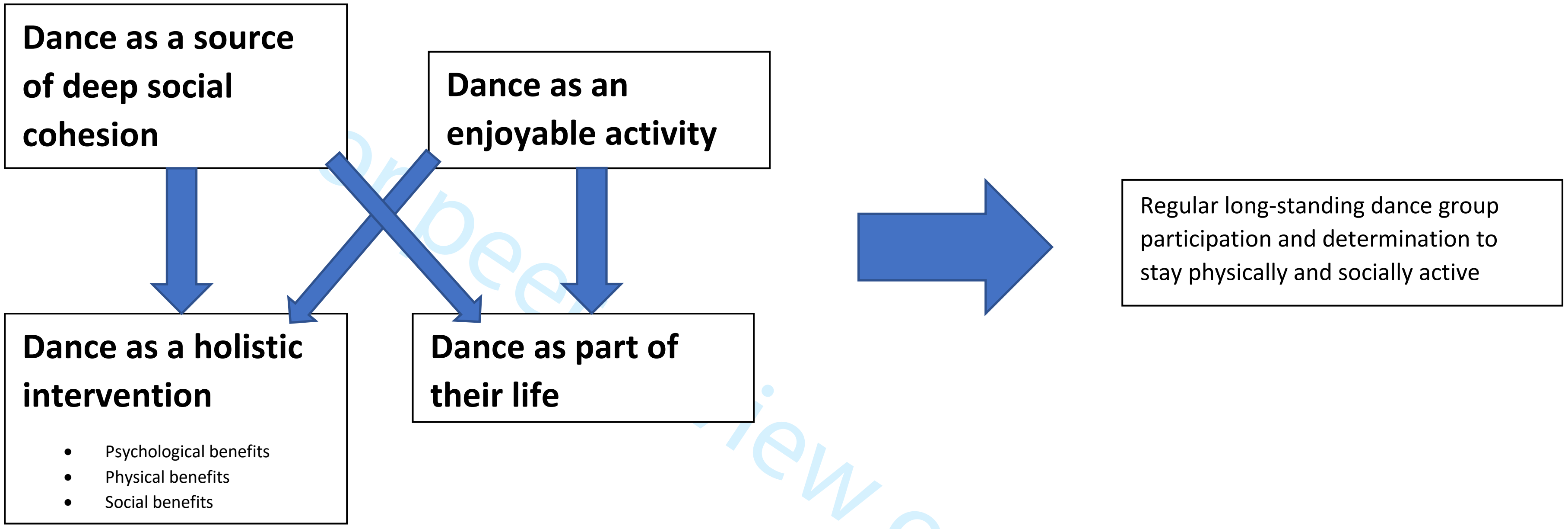

Title of Project:

Activity, Balance and Chronic Obstructive Pulmonary Disease (ABCOPD) study: Qualitative Dance Component

IRAS Project ID: 258399

Topic Guide for Interviews

Topics listed below were trialled and refined with respiratory specialist patient advisory groups prior to formal data collection.

All recordings should start with a clear statement of study participation consent in addition to the written. Initially discussion should start with rapport building with study participants. All interviews and focus groups will have audio recording on a dictaphone.

Key Topics:

How does having [primary respiratory diagnosis] affect your life?

- Symptoms
- Mood/mental health
- Socially

Physical activity (how much people do) in general life:

- What do you know about physical activity?
- What does it mean to you?
- How physically active are you?
- Has your experience of physical activity changed over time?
  -and living with lung disease?
- Should activity be avoided or actively engaged in?
- What sort of things can be done to improve physical activity levels in general?
  -and for you?

Physical capacity and physical performance (how much people can do and how well they do it)

- How would you describe the quality of your movement (in relation to stability, speed, strength and coordination?)
- Has the quality of your movement changed?
- Has your experience of your movement quality changed, for example do you find balancing, moving, or doing daily tasks more or less challenging than previously?

Experience of dance participation

How long have you been dancing?
Why do you do it?
Tell me about the dance group
How do you feel during the dance sessions?
What do you enjoy?
What do you not enjoy?

Impact of participation

What do you think are the main impacts of participating in the dance group?
Main positives?
Main negatives?
How has dancing impacted on your experience of living with lung disease?
Do you have any ideas for improving the classes?
What would you tell others who have lung disease about dancing?

Any other comments

13-FEB-19 V1

For peer review only

COREQ (COnsolidated criteria for REporting Qualitative research) Checklist

A checklist of items that should be included in reports of qualitative research. You must report the page number in your manuscript where you consider each of the items listed in this checklist. If you have not included this information, either revise your manuscript accordingly before submitting or note N/A.

Topic	Item No.	Guide Questions/Description	Reported on Page No.
**Domain 1: Research team and reflexivity**			
Personal characteristics			
Interviewer/facilitator	1	Which author/s conducted the interview or focus group?	
Credentials	2	What were the researcher's credentials? E.g. PhD, MD	
Occupation	3	What was their occupation at the time of the study?	
Gender	4	Was the researcher male or female?	
Experience and training	5	What experience or training did the researcher have?	
Relationship with participants			
Relationship established	6	Was a relationship established prior to study commencement?	
Participant knowledge of the interviewer	7	What did the participants know about the researcher? e.g. personal goals, reasons for doing the research	
Interviewer characteristics	8	What characteristics were reported about the inter viewer/facilitator? e.g. Bias, assumptions, reasons and interests in the research topic	
**Domain 2: Study design**			
Theoretical framework			
Methodological orientation and Theory	9	What methodological orientation was stated to underpin the study? e.g. grounded theory, discourse analysis, ethnography, phenomenology, content analysis	
Participant selection			
Sampling	10	How were participants selected? e.g. purposive, convenience, consecutive, snowball	
Method of approach	11	How were participants approached? e.g. face-to-face, telephone, mail, email	
Sample size	12	How many participants were in the study?	
Non-participation	13	How many people refused to participate or dropped out? Reasons?	
Setting			
Setting of data collection	14	Where was the data collected? e.g. home, clinic, workplace	
Presence of non-participants	15	Was anyone else present besides the participants and researchers?	
Description of sample	16	What are the important characteristics of the sample? e.g. demographic data, date	
Data collection			
Interview guide	17	Were questions, prompts, guides provided by the authors? Was it pilot tested?	
Repeat interviews	18	Were repeat inter views carried out? If yes, how many?	
Audio/visual recording	19	Did the research use audio or visual recording to collect the data?	
Field notes	20	Were field notes made during and/or after the inter view or focus group?	
Duration	21	What was the duration of the inter views or focus group?	
Data saturation	22	Was data saturation discussed?	
Transcripts returned	23	Were transcripts returned to participants for comment and/or	

For peer review only

Topic	Item No.	Guide Questions/Description	Reported on Page No.
		correction?	
**Domain 3: analysis and findings**			
Data analysis			
Number of data coders	24	How many data coders coded the data?	
Description of the coding tree	25	Did authors provide a description of the coding tree?	
Derivation of themes	26	Were themes identified in advance or derived from the data?	
Software	27	What software, if applicable, was used to manage the data?	
Participant checking	28	Did participants provide feedback on the findings?	
Reporting			
Quotations presented	29	Were participant quotations presented to illustrate the themes/findings? Was each quotation identified? e.g. participant number	
Data and findings consistent	30	Was there consistency between the data presented and the findings?	
Clarity of major themes	31	Were major themes clearly presented in the findings?	
Clarity of minor themes	32	Is there a description of diverse cases or discussion of minor themes?	

Developed from: Tong A, Sainsbury P, Craig J. Consolidated criteria for reporting qualitative research (COREQ): a 32-item checklist for interviews and focus groups. *International Journal for Quality in Health Care*. 2007. Volume 19, Number 6: pp. 349 – 357

Once you have completed this checklist, please save a copy and upload it as part of your submission. DO NOT include this checklist as part of the main manuscript document. It must be uploaded as a separate file.

For peer review only

BMJ Open

Dance for people with chronic respiratory disease: A qualitative study

Journal:	*BMJ Open*
Manuscript ID	bmjopen-2020-038719.R2
Article Type:	Original research
Date Submitted by the Author:	15-Sep-2020
Complete List of Authors:	Philip, Keir; National Heart and Lung Institute,<br>Lewis, Adam; Brunel University London<br>Williams, Sian; National Heart and Lung Institute<br>Buttery, Sara; National Heart and Lung Institute<br>Polkey, Michael; Royal Brompton and Harefield NHS Foundation Trust; Imperial College London<br>Man, William; Royal Brompton & Harefield, Respiratory Medicine<br>Fancourt, Daisy; University College London Research Department of Epidemiology and Public Health, Department of Behavioural Science and Health<br>Hopkinson, Nicholas; Imperial College London, National Heart and Lung Institute
**Primary Subject Heading**:	Respiratory medicine
Secondary Subject Heading:	Complementary medicine, Patient-centred medicine, Qualitative research, Rehabilitation medicine, Sports and exercise medicine
Keywords:	Adult thoracic medicine < THORACIC MEDICINE, Chronic airways disease < THORACIC MEDICINE, REHABILITATION MEDICINE, COMPLEMENTARY MEDICINE

SCHOLARONE™
Manuscripts

I, the Submitting Author has the right to grant and does grant on behalf of all authors of the Work (as defined in the below author licence), an exclusive licence and/or a non-exclusive licence for contributions from authors who are: i) UK Crown employees; ii) where BMJ has agreed a CC-BY licence shall apply, and/or iii) in accordance with the terms applicable for US Federal Government officers or employees acting as part of their official duties; on a worldwide, perpetual, irrevocable, royalty-free basis to BMJ Publishing Group Ltd ("BMJ") its licensees and where the relevant Journal is co-owned by BMJ to the co-owners of the Journal, to publish the Work in this journal and any other BMJ products and to exploit all rights, as set out in our licence.

The Submitting Author accepts and understands that any supply made under these terms is made by BMJ to the Submitting Author unless you are acting as an employee on behalf of your employer or a postgraduate student of an affiliated institution which is paying any applicable article publishing charge ("APC") for Open Access articles. Where the Submitting Author wishes to make the Work available on an Open Access basis (and intends to pay the relevant APC), the terms of reuse of such Open Access shall be governed by a Creative Commons licence – details of these licences and which Creative Commons licence will apply to this Work are set out in our licence referred to above.

Other than as permitted in any relevant BMJ Author's Self Archiving Policies, I confirm this Work has not been accepted for publication elsewhere, is not being considered for publication elsewhere and does not duplicate material already published. I confirm all authors consent to publication of this Work and authorise the granting of this licence.

For peer review only

Dance for people with chronic respiratory disease: A qualitative study

Authors: Keir EJ Philip[1] *, Adam Lewis[2], Siân Williams[1], Sara C Buttery[1], Michael I Polkey[1,3], William D-C Man[3], Daisy Fancourt[4], Nicholas S Hopkinson[1]

Affiliations:

1) National Heart and Lung Institute, Imperial College London
2) Brunel University London
3) Respiratory Medicine, Royal Brompton and Harefield NHS Foundation Trust
4) Department of Behavioural Science and Health, University College London

*Corresponding author

Author Email Addresses

- Keir Philip: k.philip@imperial.ac.uk
- Adam Lewis: Adam.Lewis@brunel.ac.uk
- Siân Williams: sian.health@gmail.com
- Sara Buttery: s.buttery@rbht.nhs.uk
- Michael Polkey: m.polkey@rbht.nhs.uk
- William Man: w.man@rbht.nhs.uk
- Daisy Fancourt: d.fancourt@ucl.ac.uk
- Nicholas Hopkinson: n.hopkinson@imperial.ac.uk

Key Words: respiratory, dance, breathlessness, physical activity, rehabilitation.

Manuscript word count: 4232 (including quotes)

For peer review only

Abstract

Objectives: To explore the experiences and perceived impact on health and well-being related to participation in a dance group for people with chronic respiratory disease (CRD).

Design: An exploratory qualitative study using thematic analysis of semi-structured interviews.

Setting: A community dance group in a UK health centre.

Participants: Convenience sample of long-term dance group participants.

Intervention: Weekly community dance sessions designed for people with breathlessness, lasting 75 minutes, led by a trained community dance leader.

Results: Convenience sample of eight participants, 6 females, aged 57 to 87 years (mean 75), with a median 2-year attendance at weekly dance sessions. Long-term attendance was driven by strongly held beliefs regarding the health and well-being benefits of participation. Four key themes were identified: Dance as (1) a holistically beneficial activity, with physical and psychosocial health benefits including improved or maintained physical fitness and psychological well-being, and reduced need for healthcare; (2) An integral part of their life; (3) An enjoyable activity; and (4) a source of deep social cohesion.

Conclusions: Dance group participants perceived a broad range of health benefits of relevance to the biopsychosocial impacts of their respiratory disease. The themes identified are useful in the ongoing planning and evaluation of dance as a holistic complex intervention for people with CRD. Further research is required to assess the extent of health impacts identified, and how dance might be most effectively placed as an option in the management of CRD.

Registered as a component of the ABCOPD study at the NIH U.S. National Library of Medicine ClinicalTrials.gov: NCT04006015

For peer review only

Article Summary: Strengths and Limitations of this study

- The first study of long-term real-world participants of a community dance group for people with chronic respiratory disease and breathlessness.
- In depth exploration of participant experience.
- The sample was from a single group which may limit transferability.
- Semi-structured interviews facilitated a depth of understanding of clinically relevant topics, aiding the potential application of findings

Original Protocol/topic guide

See Appendix 1.

For peer review only - http://bmjopen.bmj.com/site/about/guidelines.xhtml

Funding statement

KP was supported by the Imperial College Clinician Investigator Scholarship. DF was supported by the Wellcome Trust [205407/Z/16/Z]. The funders had no say in the design and conduct of the study; collection, management, analysis, and interpretation of the data; preparation, review, or approval of the manuscript; and decision to submit the manuscript for publication.

Competing Interests

Siân Williams is the founder of the dance group and leads the sessions. She was not involved in the thematic analysis.

No other conflicts of interest declared.

Authors Contributions

KP had the original idea for the study, led the study including design and ethical approval, and conducted and transcribed the interviews. KP and AL conducted the analysis and wrote the first draft of the manuscript. SW helped develop the study concept and focus, the content of the topic guide (Appendix 1), and reviewed and suggested improvements to manuscript drafts. SB, MP, WM, DF and NH provided valuable input in developing the methodological approach, developing the protocol, gaining ethical approval. All authors (KP, AL, SW, SB, MP, WM, DF, NH) contributed to the study design, writing, reviewing and editing the manuscript, and approved the final manuscript for submission.

Reporting Checklist

See attached COREQ checklist.

Data sharing statement

Data from this study are not being made available for sharing as it would not be possible to anonymise the interview transcripts given the personal content included and the very limited number of Dance groups for respiratory conditions in London.

For peer review only

Introduction

Chronic respiratory diseases (CRD) are leading causes of global morbidity and mortality (1), often causing persistent and disabling symptoms including breathlessness, cough, skeletal muscle impairment and exercise limitation (2, 3). Additionally, psychosocial impacts such as depression, loneliness and social isolation are common (4, 5) and are associated with worse clinical outcomes such as hospital admission for respiratory disease (6) and poorer physical performance (7). Physical activity, exercise training and pulmonary rehabilitation (PR) are core components of CRD management (8, 9). However, engagement is inhibited by symptoms, availability, accessibility and attractiveness of local options (10-12). Recently, interest in alternative forms of physical activity and exercise training has increased including Tai Chi (13, 14), Yoga (15) and Singing for Lung Health (SLH) (16-20). Furthermore, the current focus on social prescribing means such approaches are of increasing interest (21). Though an extensive body of research suggests arts interventions can improve health and wellbeing (22, 23), respiratory specific arts-in-health research is currently limited. Nevertheless, evidence which exists suggests a range of biopsychosocial benefits (24, 25).

Dance has been shown to be beneficial in healthy people and individuals with a range of medical conditions. Impacts include improved physical performance, function, mood, and social engagement, which are all highly relevant for people with CRD (26-33). Furthermore, dance is a core aspect of societies and cultures globally, hence has great potential as an enjoyable, engaging intervention in respiratory care throughout the world (34-36). Regarding dance for people with CRD, two feasibility studies found dance for CRD interventions to be feasible (37, 38), one also suggested dance may improve functional capacity, balance, anxiety and depression, physical activity, and health-related quality of life (38). However, no studies have assessed participants' experiences and perceptions regarding health and wellbeing impacts, which is important given the need to understand the potential of dance for wider application and how to implement and evaluate it in trials and clinical practice.

The objective of this study was to explore the experiences and perceived health and well-being impacts of participation in a community-based dance group for people with CRD - 'Dance Easy'.

Methods

Research design

We performed an exploratory qualitative study using thematic analysis within a critical realist paradigm, to explore the perceptions of participants of a community dance group for people with CRD. The primary focus was their views on the impact of participation on their health and wellbeing.

We used semi-structured interviews (see topic guide Appendix 1), developed by reviewing research on the impacts of conceptually related interventions such as PR, SLH and Tai-Chi for people with CRD. This contributed deductive elements to the analysis. However, analysis was primarily inductive using open-ended questions and open coding. Participants were encouraged to continue discussing topics which appeared meaningful with prompts. Interviews were complemented by structured observations by KP, who participated by dancing with the group over 4 sessions. This participation aimed to build rapport and trust with participants to facilitate interviews, and make structured observations to contextualise interview content, and triangulate themes. Additionally, this method

For peer review only - http://bmjopen.bmj.com/site/about/guidelines.xhtml

enabled greater reflexivity within the analysis. Participants were made aware that KP is a clinician (but not involved in their clinical care) with experience of community dance groups.

The eight regular group participants were approached face-to-face by the group leader, and all consented to participate (convenience sample) in the study. Eligibility criteria included a clinical diagnosis of CRD, age >18 years and able to consent. All participants received participant information sheets and provided signed informed consent. Ethical approval was granted by the Office for Research Ethics Committees Northern Ireland (ORECNI) (19/NI/0073).

Dance Group intervention

The group started in May 2017, with seven of the study participants present from the first few months. The group meets weekly in a community centre attached to a health centre in London. Participants usually meet in the café from 30 minutes before the session. The sessions are free for participants, the leader volunteers her time for free, and the space is provided by Whittington Health. The original participants were attendees at a 'Breathe Easy' support group for people with respiratory conditions, additional members became aware of the dance group through word of mouth.

Sessions last approximately 75 minutes and include a warmup, including qigong movements (see https://www.nqa.org/what-is-qigong- for more information on qigong), progressively complex group dance routines, warm-down, stretching and relaxation. The session leader (SW) is a trained community dance group leader with extensive experience working with people with CRD. Music is played throughout the sessions and includes an eclectic mix of genres, selected by the session leader and participants, to inspire dancing at an energy level to suit the group. Each track corresponds to a specific movement routine. Dances are selected from a repertoire, with new dances added gradually. Though led by SW, participants contribute their opinions, movements and artistic expressions.

The dances are sufficiently physically demanding to induce breathlessness, though not excessively, as judged by participants. Given the group's heterogeneity regarding disease severity, age, and co-morbidities, a degree of personalisation is required. This is achieved by providing optional variations on movements, and allowing participants to pace themselves and take breaks as required. An approach that sees the participant as the expert in their own condition and physical capacities, and trusts their ability to modify their exertion accordingly. Sessions are structured yet relaxed, focusing on dance as an enjoyable activity. Short breaks between dances are included for recovery and are used creatively to talk about dance, music and other topics that emerge. After the dance session, four to six participants go to a local pub to eat and chat, which developed spontaneously and became an established routine.

Data collection

Convenience sampling was used. Interviews were conducted one-to-one, by KP (see researcher info). Reflections and impressions were documented immediately following the interviews, with memo writing continuing throughout the analytic process. Interviews were conducted privately in the room where the dance takes place, or the café, depending on participant preference. Interviews, recorded by dictaphone, lasted 24 to 43 minutes, and consisted of open-ended questions, semi-structured around the following topics:

- Experience of respiratory illness and impact on life
- Experience of dance group
- Perceived impacts of dance group participation

For peer review only - http://bmjopen.bmj.com/site/about/guidelines.xhtml

Data analysis

Interviews were transcribed verbatim by KP. KP and AL conducted thematic analysis based on the process outlined by Braun and Clarke (39) and Terry et al. (40). During phase 1, transcripts were read and re-read, and audio recordings listened to repeatedly. In phase 2, open inclusive coding of transcripts was used. This generated first cycle codes, mainly in-vivo (verbatim), descriptive, process, emotive and value items. We chose this method to enable interpretive and more qualitative elements in our approach, which we felt was appropriate given the exploratory nature of this study. Second cycle coding using the context from the first cycle, then focused on discussions relating to dance, activity and their disease. Code mapping facilitated grouping of codes. Preliminary themes (phase 3) were constructed from code mapping and reorganisation in relation to the associated quotes. Phases 1-3 were completed by KP and AL independently, before coming together to alter, refine, consolidate and name themes (phase 4 and 5). Structured observations, reflexive accounts post-interview, and memos were incorporated to the thematic analysis. Themes were reviewed with the group leader SW. Participant respondent validation was not performed because of the value placed on researchers' interpretation within our methodology.

Demographic and disease specific information was sought. Breathlessness scores were used to indicate severity, rather than lung function measures as breathlessness relates more closely to patient related outcomes including mortality and functional impairment (2). Furthermore, participants could provide this themselves, whereas hospital investigations were not readily accessible, and participants had different CRD, which limits the relevance of spirometry group averages to this study.

Patient and Public Involvement

Patients and public were not involved in the design of the study, however the study specifically focuses on patient/participant perceptions.

Results

Eight regular dance group participants were recruited, representing the group at that time, including 6 women and 2 men, aged 57-87 years (mean 75). All reported CRD, primary diagnoses were reported as COPD (x5), asthma (x2), bronchiectasis (x1), however multimorbidity was common including arthritis, diabetes, sarcoidosis, visual impairment, and haemochromotosis. Four participants had an MRC (Medical Research Council) breathlessness scores of 2, one had a score of 3, three had scores of 1. None used supplementary oxygen. Participants lived in houses (6) and flats (2). Half of the participants lived alone, the others co-habited with their partner or children. Interviews lasted a mean of 29 minutes 46 seconds (range 23mins 47secs, to 43mins 14secs). Verbatim quotes are provided below to illustrate the themes. To facilitate understanding, explanations have also been provided in square brackets for verbatim terms to that could be unfamiliar to non-native English speakers.

No adverse events were reported. Participants perceived a wide range of health and wellbeing benefits related to attendance. All were extremely positive about the group, none expressed significant negatives, though some experienced transport related challenges. Attendance was very good, both self-reported and according to the leader's register. Non-attendance was related to holidays, transport difficulties or health issues such as optician appointments. Participants showed marked determination regarding being physically active, including but not limited to dance (walking and gardening frequently mentioned), so that '*psychologically the illness has not taken over*' (participant 5). They perceived sedentarism as highly negative, often stating the only choice being to

For peer review only - http://bmjopen.bmj.com/site/about/guidelines.xhtml

'*get on with it*' (participant 2). This determination and commitment was driven by the identified themes, which were dance as (1) A holistically beneficial activity; (2) An important part of their lives; (3) An enjoyable activity; and (4) A source of deep social cohesion. Themes 3 and 4 were drivers of themes 1 and 2, but also stood alone so were not considered subthemes (see Figure 1.

Figure 1: Thematic Map

Dance as a holistically beneficial activity

Participants viewed the sessions as a combined exercise, dance, and social activity. They described physical, psychological and social benefits, which were synergistic and, largely inseparable from one another.

> 'it's a bit of everything, afternoon social activity, because everyone has tea when they finish, or pub. It's a bit of exercise, a bit of, entertainment, it's a bit of, overall, learning.' (participant 6)

Participation was perceived as beneficial for overall health status - *'I feel like it's been a great benefit for me. It has impacted on me.'* (participant 3). Impacts were described as improvements *'you're able to do things now'* (participant 3), or physical and cognitive maintenance in the context of ageing:

> '…keeping one's body and one's mind active is very important. And that is really the be all of the end all [most important thing]. That's the object of the exercise' (participant 2), 'it helps you maintain a standard as it were, of operation in the world I suppose' (participant 2)
>
> And
>
> 'Also when the music comes on, you, about three beats and you're like, 'oh', so I like that because you're like, it's about maintaining that brain, that grey brain matter, yeah. And basically keeping you active.' (participant 6)

Perceived physical benefits included impacts on the disease and symptoms *'until I was doing the exercise it was bad'* (participant 7), and *'I think my breathing is better'* (participant 1). And implications for physical function '*mobility, I think, it's one of the things that I have found so much better. Because when I joined there were lots of things I found really difficult*' (participant 1).

Perceived mood and cognition benefits were also noted. For example, improvements in confidence,

> 'You feel you can do more things. So, if there's an offer coming up for an outing to a garden all day, I know that I'm going to go' (participant 4)

And,

> 'Psychologically the illness has not taken over… because you're occupying your mind on something completely different, so you can't say ' oh I can't do that, I can't breathe', I can have a go. That's what life's all about at the end of the day' (participant 5)

Impacts on mood were driven by enjoying participation (theme 3) and a sense of the group's deep social cohesion (theme 4), see figure 1 (thematic map). Additionally, participants 1, 3 and 5 perceived these holistic health benefits as leading to reduced healthcare utilisation, *'I haven't had a bad*

For peer review only - http://bmjopen.bmj.com/site/about/guidelines.xhtml

bronchitis episode since I've been doing it' (participant 1), and *'you're not so prone to picking up all these chest infections'* (participant 3).

Dance as an important part of their lives

Dance was perceived as an important part of their lives, *'It's just part of my life now'* (participant 3). For some participants dance had been important throughout their lives (all but one reported dancing 'socially' at family events, 3 took classes), while for others, dance only gained importance since joining the group *'I was your typical wallflower …. I never really got into dancing at all. Till now.'* (participant 3).

In general, the importance of dance participation was not related to their perceived ability or previous experience - *'I wouldn't call myself twinkle toes [a good dancer]'* (participant 3) and *'I can't dance for toffee [I can't dance well], and I can't sing, but hey (laughs) who cares.'* (participant 1). However, perceptions regarding dance ability influenced how participants described what they do during the sessions. For example, some participants commented that they were not really dancing as they felt that a certain skill level was required for their individual definition of dance, instead describing the sessions as '*movement to music'* (participant 2). Nevertheless, learning to dance was frequently stated as a component of the sessions, hence, differing definitions of dance likely represented differing life experiences of dance, rather than differing views on what they gained from participation in the group.

> 'I'm learning something new and it's also, you come in and learn a dance that six weeks ago you never thought you'd get your feet around [never thought you could learn the dance steps].' (participant 6)

This satisfaction related to a sense of mastery was also important. Observation data also noted participants improved over the sessions, particularly completing routines with fewer errors, with a clear sense of achievement-related satisfaction, perhaps as a contrast to a background of decline in other aspects of their functioning. Structured observations also noted that participants were often quite elegantly dressed, which may not be expected in activities seen as purely exercise.

Dance as an enjoyable activity

All participants greatly enjoyed the sessions, consistently describing it as fun, which was a main driver of attendance. This was repeatedly emphasised as being of major importance, and something participants felt was not appreciated or considered in other therapeutic exercise they had attended. In this sense dancing was viewed as an end-in-itself, rather than the means to an end, as was the case for gym-based exercise training. Multiple factors contributed to their enjoyment including a light-hearted and non-judgemental atmosphere, in which participants felt active contributors rather than passive recipients of care.

> 'There is this immense feeling of contentment, and it's great. You know, when you've made an effort, you've done it, and jolly good. And that's it.' (participant 2)

The atmosphere in the sessions was very relaxed. The element of humour was highlighted in structured observations, with regular group laughter noted, with a very inclusive quality.

> '…before you know it, we're all in stiches [all laughing]. You know it's something, you know. Its promoted in the group. You can feel it. You get the buzz. You have a good laugh and, apart from doing your dancing you're having fun, even with the dancing you're having fun.' (participant 3)

For peer review only - http://bmjopen.bmj.com/site/about/guidelines.xhtml

Not feeling intimidated or judged was key to the enjoyment and contrasted with other exercise modalities and settings

> 'I won't go to the gym where there's a lot of younger people than myself. Because they can be intimidating. So, I won't do nothing like that.' (participant 5)

Dance as a source of deep social cohesion

Participants described the other group members in very affectionate terms – *'I love everyone I'm with', 'we're like a small knit family [close social relationship, care for each other like a family]'* (participant 6), and greatly valued their relationships with other individuals and the group as a whole.

> 'It's not just about dancing it's about the whole, being part of the organisation, being part of a little unit that I think I enjoy coming out.' (participant 6)

Having shared experience was extremely important to participants. Participants commented on feeling 'other' when in public or with friends outside of the group. Paradoxically, although the group is built around having respiratory disease, not feeling 'other' created an environment where the disease could be forgotten, providing a form of respite.

> 'I do it for the social side as well. Because it's very nice to meet people with the same basic difficulties' (participant 2)

> 'As a group, you know, we obviously bonded with each other, well we know each other, it is, you know it's something that…. I think we all enjoy it.' (participant 3)

> 'we kind of understand each other when we can't breathe' (participant 6)

> 'But I think that for me it's about forgetting what's wrong with me until I actually go home.' (participant 6)

The depth of connection between participants underpinned the holistic impacts described in theme 1. The shared experience enabled participants to develop a combination of acceptance and increased motivation for improvement. This forged a collective self-motivation, with participants seeing their health as their own responsibility, directly related to the effort they put in *'you got to be up for it, you've got to be disciplined'* (participant 4). Yet, simultaneously appreciating that this could be best achieved together.

> 'for me it's a good thing. It's basically being with likeminded people, people who understand how you feel. You can see them, you can encourage them to do a little better. I think I've actually changed my attitude. Instead of being in denial about not being able to breathe, I think I've more embraced it.' (participant 6)

This deep social cohesion was attributed to the content of the sessions, particularly the presence of music, and dancing together.

> 'Well it means you accept the whole thing. You're moving with a group of people who have a similar problem.' (participant 2)

The supportive atmosphere was also attributed to session leader SW. SW, is very supportive while challenging the participants to do the best they can, which is greatly appreciated by the participants who describe her as 'a lovely person'. There is a clear focus on abilities rather than impairment, which helps set the tone for the entire group.

For peer review only - http://bmjopen.bmj.com/site/about/guidelines.xhtml
For peer review only

'...the wonderful thing is, nobody's ever been told "you're useless and you can't do it' (participant 1)

(referring to the group leader's responses) 'It's the effort that one puts into it, and that is repaid enormously' (participant 2)

The social relationships that have developed were also contrasted with other types of therapeutic exercise such as going to the gym, where they felt this depth of relationship was not created.

'if you go to the gym, you're just doing your own thing, whereas here we are supporting each other' (participant 1)

Discussion

This study found that long-standing participants in a dance group for people with CRD perceived a range of holistic health and wellbeing benefits. The dance group had become an important part of their lives. Enjoyment of participation and the development of deep social cohesion between participants were considered key drivers of these impacts.

This study has multiple strengths. To our knowledge it is the first study to explore community dance participation for individuals with respiratory disease in a real-world setting. A recent pilot study included qualitative aspects, based on informal group discussions and field notes (37), but no other studies have used in-depth qualitative interviews with long-term attendees, out-side of a trial setting, which improves the relevance of our work to real-world experience. Indeed, few qualitative studies of arts-in-health interventions in respiratory disease have explored such long-term participants' experiences. Secondly, the use of semi-structured interviews enabled inductive exploration of specific topics of interest. Thirdly, interpretation of the interview data was strengthened though use of structured observations.

Some limitations should be noted. Firstly, sample size was limited by the number of group participants. However, given the exploratory nature of the study, sufficient data were collected to justify the themes. Secondly, as we only focused on a single group, the transferability of findings cannot be ascertained. Thirdly, more detailed clinical characterisation of the participants' respiratory disease may have been of interest to people reading this study if considering practical implications for a specific context. However, we collected the relevant data for our research question. Fourthly, a degree of selection bias may exist, as study participants were individuals who had maintained attendance, while those that did not enjoy the group no longer participated and were not included in the research. It would have been useful to interview people who no longer attended to explore the reasons for non-attendance. Similarly, the group has a small number of participants, which is likely due to no formal promotion activities because it is unfunded. Queries about the group are managed by the Breathe Easy team and no formal referral pathway yet exists. It is also possible the small group size maybe small because potential participants did not see this as a desirable activity, but without further research it is not possible to draw any definite conclusions.

Our findings echo some of the findings from the limited existing research regarding dance for people with CRD. A recent study assessing a dance intervention for COPD showed it to be feasible, with secondary outcome measures suggesting improvements in functional capacity, balance, anxiety and depression, physical activity, and health-related quality of life (38). These findings suggest that elements of the perceived holistic benefits described in our study have quantifiable impacts using established assessment tools. Additionally, a second feasibility study assessing dance for people with breathlessness reported preliminary results suggesting dancing together is of importance, and

For peer review only - http://bmjopen.bmj.com/site/about/guidelines.xhtml

participants reported 'coming alive' (37). Such findings appear similar to themes three and four of our study. The themes identified here also map to multiple levels of Maslow's hierarchy of needs model of human motivation (41) including safety, love and belonging, self-esteem, and self-actualisation. This supports our interpretation, that their experience as being complex and holistically beneficial, drove their motivation for participation. Overall, our findings are broadly corroborative with existing studies, while deepening understanding of underlying drivers for participation and psychological benefits observed.

Research exploring the experiences of people with CRD participating in related activities shows some similarities and differences. Regarding pulmonary rehabilitation (PR), a high-value intervention(42) which commonly includes group based callisthenic type exercises, group support and increased self-confidence have been found to promote adherence (43). However, lack of social support and overcoming the effort of living with COPD, as well as anxiety and depression (11) have been identified as negatively influencing participation in PR (43). The participants in our study showed consistent attendance over an extended period, despite often living alone or reporting limited social contacts. Potential explanations include the dance group providing an experience sufficiently different from PR to overcome these issues, with participants reporting strong social connectiveness with peers, and high levels of confidence in their abilities, despite the evident disease-based limitations. A related activity is SLH which consists of specially developed group singing sessions for people with CRD. Research on SLH has found very similar impacts to the current study, including positive impacts on general wellbeing, community/social support and enjoyment of participation. Both suggest physical benefits (17-19). Our findings echo those of research on community dance interventions in different patient groups including improving physical performance, mood, cognition, quality of life (31, 44, 45) sense of joy and pride (46), interpersonal relationships and connections (47). Hence, the perceived health impacts here reported may not be disease specific, but rather represent the impact of group dance participation on people in general. That said, given the biopsychosocial impacts of CRD and relative lack of effective management options for psychosocial components, dance interventions appear particularly well suited for people with CRDs.

Of note, the study participants did not report severe breathlessness, none used supplementary oxygen, and all lived relatively independently. Given the strong evidence base for pulmonary rehabilitation and other forms of exercise in people with more severe breathlessness and disease related impairment, it would be of interest to explore the use of dance interventions in such individuals. Similarly, they were generally quite active people, who clearly valued various types of physical activity, this may frame their highly positive perspectives, and indicates further research on interventions targeting less-active individuals would be of value, as well as groups who meet via the internet.

Conclusion

CRDs are common and result in a high burden of morbidity, mortality and related economic implications. Therefore, low-cost, low-resource interventions with holistic impacts on health and wellbeing should be of broad interest. In particular, the perceived impacts on physical performance, psychosocial health, and health resource utilisation are promising. Furthermore, this study highlights the importance of developing enjoyable physical activities that provide opportunity for self-expression for people with CRD to utilise intrinsic motivation, as an end in itself, but also to support compliance.

Participants perceived a broad range of positive holistic health and wellbeing benefits; viewed participation as an important part of their lives; greatly enjoyed participation; and felt deep social

For peer review only - http://bmjopen.bmj.com/site/about/guidelines.xhtml

cohesion with other participants. Further research is required to confirm, clarify and quantify these impacts, and assess the cost effectiveness of sustainable methods of delivery including funding for session leaders and an appropriate dance space'. .

Information on lead researcher:

Dr Keir Philip (KP) is a male, 33-year-old, respiratory physician who previously worked as a dancer and dance teacher, with experience leading community dance and dance for people with respiratory disease. KP has received training in qualitative research methods from the Imperial College, University College London and through self-directed learning. He is currently completing a PhD at Imperial College, using both qualitative and quantitative methods. Qualifications: MBChB BSc MRCP EADTMH DPMSA.

KP and AL are dancers recreationally and previous dance experiences enabled discussions throughout the analysis to be explored fully and assumptions challenged within the researchers' assumptions of what dance is.

Acknowledgements

We would like to give our thanks to the study participants for their time and effort.

For peer review only

For peer review only - http://bmjopen.bmj.com/site/about/guidelines.xhtml

References

1. Ferkol T, Schraufnagel D. The global burden of respiratory disease. Annals of the American Thoracic Society. 2014;11(3):404-6.
2. Kelly JL, Bamsey O, Smith C, Lord VM, Shrikrishna D, Jones PW, et al. Health status assessment in routine clinical practice: the chronic obstructive pulmonary disease assessment test score in outpatients. Respiration. 2012;84(3):193-9.
3. Nishiyama O, Taniguchi H, Kondoh Y, Kimura T, Ogawa T, Watanabe F, et al. Quadriceps weakness is related to exercise capacity in idiopathic pulmonary fibrosis. Chest. 2005;127(6):2028-33.
4. Gardiner C, Gott M, Payne S, Small N, Barnes S, Halpin D, et al. Exploring the care needs of patients with advanced COPD: an overview of the literature. Respir Med. 2010;104(2):159-65.
5. Yohannes AM, Alexopoulos GS. Depression and anxiety in patients with COPD. Eur Respir Rev. 2014;23(133):345-9.
6. Bu F, Philip K, Fancourt D. Social isolation and loneliness as risk factors for hospital admissions for respiratory disease among older adults. Thorax. 2020.
7. Philip KEJ, Polkey MI, Hopkinson NS, Steptoe A, Fancourt D. Social isolation, loneliness and physical performance in older-adults: fixed effects analyses of a cohort study. Scientific reports. 2020;10(1):13908.
8. Spruit MA, Singh SJ, Garvey C, ZuWallack R, Nici L, Rochester C, et al. An official American Thoracic Society/European Respiratory Society statement: key concepts and advances in pulmonary rehabilitation. American journal of respiratory and critical care medicine. 2013;188(8):e13-64.
9. Hopkinson NS, Molyneux A, Pink J, Harrisingh MC. Chronic obstructive pulmonary disease: diagnosis and management: summary of updated NICE guidance. BMJ. 2019;366:l4486.
10. Rochester CL, Vogiatzis I, Holland AE, Lareau SC, Marciniuk DD, Puhan MA, et al. An Official American Thoracic Society/European Respiratory Society Policy Statement: Enhancing Implementation, Use, and Delivery of Pulmonary Rehabilitation. American journal of respiratory and critical care medicine. 2015;192(11):1373-86.
11. Boutou AK, Tanner RJ, Lord VM, Hogg L, Nolan J, Jefford H, et al. An evaluation of factors associated with completion and benefit from pulmonary rehabilitation in COPD. BMJ Open Respir Res. 2014;1(1):e000051.
12. Philip K, Gaduzo S, Rogers J, Laffan M, Hopkinson NS. Patient experience of COPD care: outcomes from the British Lung Foundation Patient Passport. BMJ open respiratory research. 2019;6(1):e000478.
13. Polkey MI, Qiu Z-H, Zhou L, Zhu M-D, Wu Y-X, Chen Y-Y, et al. Tai Chi and Pulmonary Rehabilitation Compared for Treatment-Naive Patients With COPD: A Randomized Controlled Trial. Chest. 2018;153(5):1116-24.
14. Lewis A, Hopkinson NS. Tai Chi Movements for Wellbeing – evaluation of a British Lung Foundation pilot. Perspectives in Public Health. 2019:1757913919872515.
15. Nolan CM, Rochester CL. Exercise Training Modalities for People with Chronic Obstructive Pulmonary Disease. Copd. 2019;16(5-6):378-89.
16. Lewis A, Cave P, Hopkinson NS. Singing for Lung Health: a qualitative assessment of a British Lung Foundation programme for group leaders. BMJ open respiratory research. 2017;4(1):e000216.
17. Lewis A, Cave P, Hopkinson NS. Singing for Lung Health: service evaluation of the British Lung Foundation programme. Perspect Public Health. 2018:1757913918774079.
18. Lewis A, Cave P, Stern M, Welch L, Taylor K, Russell J, et al. Singing for Lung Health-a systematic review of the literature and consensus statement. NPJ primary care respiratory medicine. 2016;26:16080.
19. Lord VM, Cave P, Hume VJ, Flude EJ, Evans A, Kelly JL, et al. Singing teaching as a therapy for chronic respiratory disease--a randomised controlled trial and qualitative evaluation. BMC pulmonary medicine. 2010;10:41.

For peer review only - http://bmjopen.bmj.com/site/about/guidelines.xhtml

20. Lord VM, Hume VJ, Kelly JL, Cave P, Silver J, Waldman M, et al. Singing classes for chronic obstructive pulmonary disease: a randomized controlled trial. BMC pulmonary medicine. 2012;12:69.
21. Drinkwater C, Wildman J, Moffatt S. Social prescribing. Bmj. 2019;364:l1285.
22. Fancourt D, Finn S. What is the evidence on the role of the arts in improving health and well-being? A scoping review. World Health Organisation; 2019.
23. Fancourt D, Steptoe A. The art of life and death: 14 year follow-up analyses of associations between arts engagement and mortality in the English Longitudinal Study of Ageing. BMJ. 2019;367:l6377.
24. Philip K, Lewis A, Hopkinson NS. Music and dance in chronic lung disease. Breathe. 2019;15(2):116-20.
25. Philip KEJ, Williams, S., Simpson, J., Rooney, C., Davey, C., Gupta, R., Singh, R.,. Exploring participants current experiences and opinions regarding use of music/dance in pulmonary rehabilitation. European Respiratory Society Conference; Milan, Italy. European Respiratory Journal2017.
26. Fong Yan A, Cobley S, Chan C, Pappas E, Nicholson LL, Ward RE, et al. The Effectiveness of Dance Interventions on Physical Health Outcomes Compared to Other Forms of Physical Activity: A Systematic Review and Meta-Analysis. Sports Med. 2018;48(4):933-51.
27. Hwang PW, Braun KL. The Effectiveness of Dance Interventions to Improve Older Adults' Health: A Systematic Literature Review. Altern Ther Health Med. 2015;21(5):64-70.
28. Jeong YJ, Hong SC, Lee MS, Park MC, Kim YK, Suh CM. Dance movement therapy improves emotional responses and modulates neurohormones in adolescents with mild depression. Int J Neurosci. 2005;115(12):1711-20.
29. Merom D, Ding D, Stamatakis E. Dancing Participation and Cardiovascular Disease Mortality: A Pooled Analysis of 11 Population-Based British Cohorts. American journal of preventive medicine. 2016;50(6):756-60.
30. Murrock CJ, Graor CH. Effects of dance on depression, physical function, and disability in underserved adults. J Aging Phys Act. 2014;22(3):380-5.
31. Patterson KK, Wong JS, Prout EC, Brooks D. Dance for the rehabilitation of balance and gait in adults with neurological conditions other than Parkinson's disease: A systematic review. Heliyon. 2018;4(3):e00584.
32. Rodrigues-Krause J, Krause M, Reischak-Oliveira A. Dancing for Healthy Aging: Functional and Metabolic Perspectives. Altern Ther Health Med. 2019;25(1):44-63.
33. Sharp K, Hewitt J. Dance as an intervention for people with Parkinson's disease: a systematic review and meta-analysis. Neurosci Biobehav Rev. 2014;47:445-56.
34. Philip KEJ. Sing a song of lung health-beyond the UK. Bmj. 2019;364:l652.
35. Philip KE, Akylbekov A, Stambaeva B, Sooronbaev T, Jones R. Music, Dance, and Harmonicas for People With COPD. Respiratory care. 2019;64(3):359.
36. Philip KEJ, Katagira W, Jones R. Dance for Respiratory Patients in Low-Resource Settings. Jama. 2020;324(10):921-2.
37. Harrison SL BK, Edwards J, McFaull V, McLusky S, Russell A, Williams G, Williams S. DANCE FOR PEOPLE WITH CHRONIC BREATHLESSNESS: A FEASIBILITY STUDY. Thorax2019.
38. Wshah A, Butler S, Patterson K, Goldstein R, Brooks D. "Let's Boogie": FEASIBILITY OF A DANCE INTERVENTION IN PATIENTS WITH CHRONIC OBSTRUCTIVE PULMONARY DISEASE. J Cardiopulm Rehabil Prev. 2019;39(5):E14-E9.
39. Braun V, Clarke V.,. Using thematic analysis in psychology. Qualitative Research in Psychology. 2006;3(2):77-101.
40. Terry G, Hayfield, N., Clarke, V., Braun, V.,. Thematic Analysis in: The SAGE Handbook of Qualitative Research in Psychology. London: SAGE Publications Ltd; 2017.
41. Maslow AH. A theory of human motivation. Psychology Review. 1943;50(4):370–96.

For peer review only

42. Zoumot Z, Jordan S, Hopkinson NS. Emphysema: time to say farewell to therapeutic nihilism. Thorax. 2014;69(11):973-5.
43. Arnold E, Bruton A, Ellis-Hill C. Adherence to pulmonary rehabilitation: A qualitative study. Respir Med. 2006;100(10):1716-23.
44. Eyigor S, Karapolat H, Durmaz B, Ibisoglu U, Cakir S. A randomized controlled trial of Turkish folklore dance on the physical performance, balance, depression and quality of life in older women. Arch Gerontol Geriatr. 2009;48(1):84-8.
45. Granacher U, Muehlbauer T, Bridenbaugh SA, Wolf M, Roth R, Gschwind Y, et al. Effects of a salsa dance training on balance and strength performance in older adults. Gerontology. 2012;58(4):305-12.
46. Alfredsson Olsson E, Heikkinen S. "I will never quit dancing". The emotional experiences of social dancing among older persons. J Aging Stud. 2019;51:100786.
47. Atkins R, Deatrick JA, Gage GS, Earley S, Earley D, Lipman TH. Partnerships to Evaluate the Social Impact of Dance for Health: A Qualitative Inquiry. J Community Health Nurs. 2019;36(3):124-38.

For peer review only

For peer review only - http://bmjopen.bmj.com/site/about/guidelines.xhtml

Thematic Map

A thematic map showing the main four themes and the key ways they relate to one another.

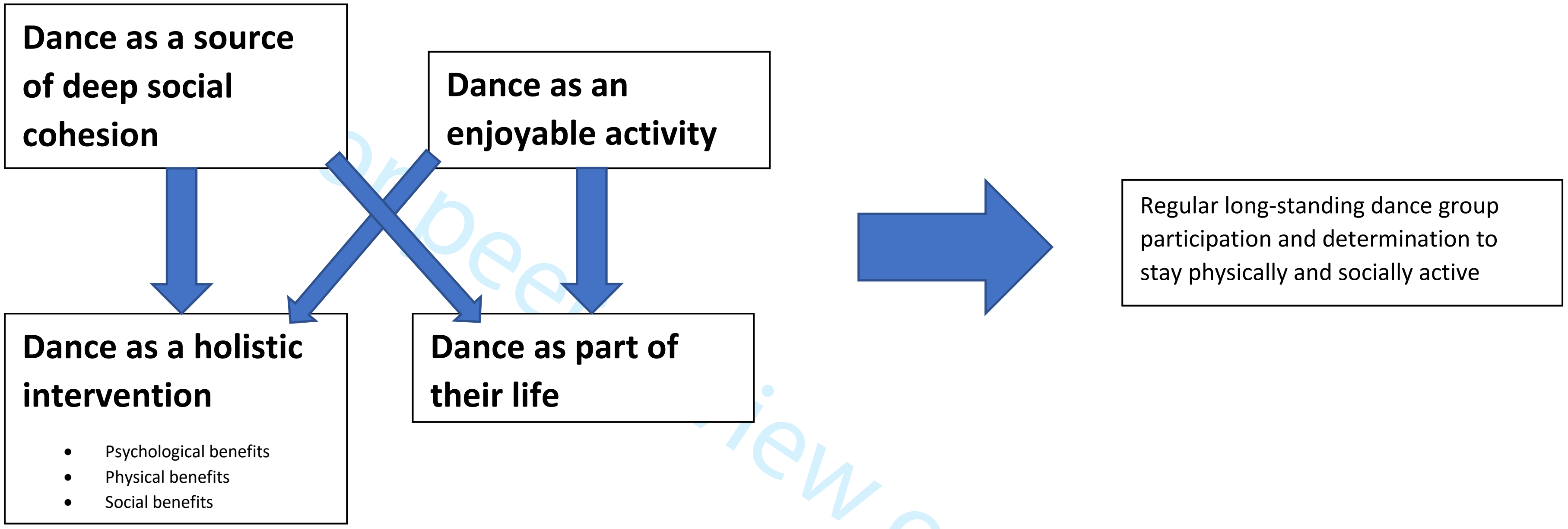

Title of Project:

Activity, Balance and Chronic Obstructive Pulmonary Disease (ABCOPD) study: Qualitative Dance Component

IRAS Project ID: 258399

Topic Guide for Interviews

Topics listed below were trialled and refined with respiratory specialist patient advisory groups prior to formal data collection.

All recordings should start with a clear statement of study participation consent in addition to the written. Initially discussion should start with rapport building with study participants. All interviews and focus groups will have audio recording on a dictaphone.

For peer review only

Key Topics:

How does having [primary respiratory diagnosis] affect your life?

- Symptoms
- Mood/mental health
- Socially

Physical activity (how much people do) in general life:

- What do you know about physical activity?
- What does it mean to you?
- How physically active are you?
- Has your experience of physical activity changed over time?
  -and living with lung disease?
- Should activity be avoided or actively engaged in?
- What sort of things can be done to improve physical activity levels in general?
  -and for you?

Physical capacity and physical performance (how much people can do and how well they do it)

- How would you describe the quality of your movement (in relation to stability, speed, strength and coordination?)
- Has the quality of your movement changed?
- Has your experience of your movement quality changed, for example do you find balancing, moving, or doing daily tasks more or less challenging than previously?

Experience of dance participation

How long have you been dancing?
Why do you do it?
Tell me about the dance group
How do you feel during the dance sessions?
What do you enjoy?
What do you not enjoy?

Impact of participation

What do you think are the main impacts of participating in the dance group?
Main positives?
Main negatives?
How has dancing impacted on your experience of living with lung disease?
Do you have any ideas for improving the classes?
What would you tell others who have lung disease about dancing?

Any other comments

13-FEB-19 V1

COREQ (COnsolidated criteria for REporting Qualitative research) Checklist

A checklist of items that should be included in reports of qualitative research. You must report the page number in your manuscript where you consider each of the items listed in this checklist. If you have not included this information, either revise your manuscript accordingly before submitting or note N/A.

Topic	Item No.	Guide Questions/Description	Reported on Page No.
**Domain 1: Research team and reflexivity**			
Personal characteristics			
Interviewer/facilitator	1	Which author/s conducted the interview or focus group?	
Credentials	2	What were the researcher's credentials? E.g. PhD, MD	
Occupation	3	What was their occupation at the time of the study?	
Gender	4	Was the researcher male or female?	
Experience and training	5	What experience or training did the researcher have?	
Relationship with participants			
Relationship established	6	Was a relationship established prior to study commencement?	
Participant knowledge of the interviewer	7	What did the participants know about the researcher? e.g. personal goals, reasons for doing the research	
Interviewer characteristics	8	What characteristics were reported about the inter viewer/facilitator? e.g. Bias, assumptions, reasons and interests in the research topic	
**Domain 2: Study design**			
Theoretical framework			
Methodological orientation and Theory	9	What methodological orientation was stated to underpin the study? e.g. grounded theory, discourse analysis, ethnography, phenomenology, content analysis	
Participant selection			
Sampling	10	How were participants selected? e.g. purposive, convenience, consecutive, snowball	
Method of approach	11	How were participants approached? e.g. face-to-face, telephone, mail, email	
Sample size	12	How many participants were in the study?	
Non-participation	13	How many people refused to participate or dropped out? Reasons?	
Setting			
Setting of data collection	14	Where was the data collected? e.g. home, clinic, workplace	
Presence of non-participants	15	Was anyone else present besides the participants and researchers?	
Description of sample	16	What are the important characteristics of the sample? e.g. demographic data, date	
Data collection			
Interview guide	17	Were questions, prompts, guides provided by the authors? Was it pilot tested?	
Repeat interviews	18	Were repeat inter views carried out? If yes, how many?	
Audio/visual recording	19	Did the research use audio or visual recording to collect the data?	
Field notes	20	Were field notes made during and/or after the inter view or focus group?	
Duration	21	What was the duration of the inter views or focus group?	
Data saturation	22	Was data saturation discussed?	
Transcripts returned	23	Were transcripts returned to participants for comment and/or	

For peer review only

Topic	Item No.	Guide Questions/Description	Reported on Page No.
		correction?	
**Domain 3: analysis and findings**			
Data analysis			
Number of data coders	24	How many data coders coded the data?	
Description of the coding tree	25	Did authors provide a description of the coding tree?	
Derivation of themes	26	Were themes identified in advance or derived from the data?	
Software	27	What software, if applicable, was used to manage the data?	
Participant checking	28	Did participants provide feedback on the findings?	
Reporting			
Quotations presented	29	Were participant quotations presented to illustrate the themes/findings? Was each quotation identified? e.g. participant number	
Data and findings consistent	30	Was there consistency between the data presented and the findings?	
Clarity of major themes	31	Were major themes clearly presented in the findings?	
Clarity of minor themes	32	Is there a description of diverse cases or discussion of minor themes?	

Developed from: Tong A, Sainsbury P, Craig J. Consolidated criteria for reporting qualitative research (COREQ): a 32-item checklist for interviews and focus groups. *International Journal for Quality in Health Care*. 2007. Volume 19, Number 6: pp. 349 – 357

Once you have completed this checklist, please save a copy and upload it as part of your submission. DO NOT include this checklist as part of the main manuscript document. It must be uploaded as a separate file.

For peer review only
